# The effect of intravenous dexamethasone in supraclavicular brachial plexus block at Halibet National Referral Hospital, Eritrea 2024–2025: A randomized clinical trial

**DOI:** 10.1371/journal.pone.0338067

**Published:** 2026-09-03

**Authors:** Michael Beraki Mengistu, Senay Amare Habtu, Abel Mussie Elias, Sabrina Hashim Idris, Kisanet Huruy Abraha

**Affiliations:** Department of Nursing, Anesthesia Unit, Orotta College of Medicine and Health Sciences, Asmara, Eritrea; Stanford University School of Medicine, UNITED STATES OF AMERICA

## Abstract

**Background:**

Postoperative pain has significant impact on recovery and healthcare costs. While effective regional anesthesia provides both anesthesia and analgesia, its effects are often of limited duration. Previous studies indicate that intravenous dexamethasone may enhance and prolong analgesia when used with regional blocks, yet reports on its duration to supraclavicular brachial plexus block (SCBPB) for upper limb surgeries are limited. We hypothesized that intravenous dexamethasone would extend postoperative analgesia following SCBPB.

**Method:**

A randomized clinical trial was conducted at Halibet National Referral Hospital in Eritrea, involving 60 patients undergoing upper limb surgery who received SCBPB with 20 ml of 0.5% bupivacaine. Concurrently, the control group received 2 ml of normal saline, while the experimental group received 2 ml (8 mg) of intravenous dexamethasone. Pain scores, time to first rescue analgesia, and analgesic consumption were recorded. Data were analyzed using SPSS v26.

**Results:**

The mean duration of analgesia was significantly longer in the dexamethasone group (17.36 ± 3.75 hours) compared to the control group (7.57 ± 1.04 hours, p < 0.001). The dexamethasone group had faster onset of sensory and motor block and lower analgesic consumption.

**Conclusions:**

In summary, intravenous dexamethasone significantly prolonged postoperative analgesia, accelerated block onset, and reduced analgesic consumption without adverse events. These findings support its use as a practical adjunct to bupivacaine in supraclavicular brachial plexus block, with potential to improve postoperative pain management in low‑resource settings.

**Clinical trial registration:**

This trial was registered in the Pan African Clinical Trial Registry (PACTR202509579633417). Participant recruitment occurred between September 23, 2024 and January 17, 2025, with follow‑up completed by January 18, 2025. The authors confirm that all ongoing and related trials for this intervention are registered.

## Introduction

Pain is not merely a sensory modality but a multidimensional experience. The International Association for the Study of Pain (IASP) defines it as “an unpleasant sensory and emotional experience associated with actual or potential tissue damage” [1]. This definition highlights the interplay between subjective perception, emotional response, and physiological mechanisms. Pain response vary widely among individuals and even within the same person over times, influenced by factors such as age, gender and psychological state. In hospital settings, both acute and chronic pain are common, making postoperative pain management essential for all patients [1].

Despite advances in postoperative pain (POP) management, significant gaps remain. Acute POP is often undertreated, particularly following general surgical procedures. Unrelieved pain can negatively affect physical and psychological well-being, delay recovery, and increase healthcare costs. Immediate consequences of severe pain include reduced mobility, sleep disturbances, increased reliance on analgesics, and impaired immune function, which may heighten susceptibility to infection [2]. Poorly managed pain can also trigger pathophysiological changes in the peripheral and central nervous systems, potentially leading to chronic pain. In such cases, central sensitization may result in exaggerated pain responses to stimuli that would otherwise be tolerable. [3].

Regional anesthesia offers excellent analgesia and is widely accepted for POP relief [4]. For upper limb surgeries, brachial plexus block (BPB) is used either as an adjunct to general anesthesia or as a sole anesthetic technique. However, the duration of analgesia is limited by the pharmacokinetics of local anesthetics (LAs). Prolongation can be achieved through indwelling perineural catheters or the addition of adjuvant drugs. While catheter techniques provide extended analgesia, they are limited by technical challenges such as placement accuracy, risk of infection, and removal difficulties [5,6].

Recent advancements in peripheral nerve block (PNB) techniques and the use of adjuvants have significantly improved analgesic outcomes. Adjuvants—agents administered alongside LAs—enhance block quality, extend analgesia duration, and improve patient recovery [7]. A comprehensive understanding of the mechanisms of action, clinical effects, and safety profiles of PNB and the use of additional adjuvants is essential.

Brachial plexus blocks can be performed via interscalene, supraclavicular, infraclavicular, or axillary approaches. Among these, the supraclavicular route is preferred for procedures below the shoulder due to its consistency and ease of access. However, complications such as pneumothorax, hemothorax, Horner’s syndrome, and phrenic nerve block may occur. Ultrasound-guided supraclavicular blocks improve safety by allowing real-time visualization of anatomical structures and needle placement.

Although LAs alone provide good intraoperative conditions, their effects wear off within hours, exposing patients to moderate to severe postoperative pain [8]. To address this, various adjuvants—including morphine, tramadol, fentanyl, clonidine, dexmedetomidine, and midazolam—have been used. [9]. Recently, corticosteroids such as dexamethasone have gained attention for their analgesic and antiemetic properties. Dexamethasone can be administered systemically or perineurally and has shown efficacy in enhancing peripheral nerve blocks [10].

Dexamethasone is a long-acting glucocorticoid (half-life >36 hours) with potent anti-inflammatory and analgesic effects [11]. It prolongs nerve block duration by inhibiting nociceptive C-fiber transmission and modulating potassium channel activity in excitable cells [12]. Additionally, dexamethasone induces vasoconstriction, slowing local anesthetic absorption and extending its effect [13].

A single 8 mg dose of intravenous dexamethasone combined with bupivacaine in supraclavicular brachial plexus block has been shown to reduce postoperative pain intensity and prolong analgesia without adverse effects. Its low cost, antiemetic properties, and favorable safety profile make it a practical alternative to perineural administration, especially in resource-limited settings [14]. To date, no studies have investigated this approach in Eritrea, and research on this topic remains scarce. Therefore, this study was conducted to compare the duration of analgesia between two groups—one receiving bupivacaine alone and the other receiving bupivacaine with intravenous dexamethasone—in supraclavicular brachial plexus blocks.

We hypothesized that intravenous dexamethasone, when combined with bupivacaine in supraclavicular brachial plexus block, would prolong postoperative analgesia, accelerate the onset of sensory and motor block, and reduce analgesic consumption compared to bupivacaine alone. This hypothesis is based on prior evidence of corticosteroids’ analgesic and anti‑inflammatory properties, as well as their ability to modulate nociceptive transmission and prolong block duration.

## Materials and methods

### Study design and setting

This study was a hospital-based, randomized clinical trial designed to evaluate the effect of intravenous dexamethasone combined with bupivacaine on prolonging postoperative analgesia following supraclavicular brachial plexus block (SCBPB). It was conducted in the orthopedic department of Halibet National Referral Hospital (HNRH), the sole government-run tertiary center providing orthopedic services in Eritrea. The hospital is located in Asmara, the capital city.

### Study participants

Sixty adult patients scheduled for elective upper limb surgeries under SCBPB were enrolled. Inclusion criteria were: age between 18 and 80 years, ASA physical status I or II, and procedures involving the elbow, forearm, or hand. Exclusion criteria included: patient refusal, contraindications to SCBPB (e.g., severe chronic obstructive pulmonary disease, contralateral diaphragmatic paralysis, neuropathy of the surgical limb), pregnancy, bleeding disorders, local infection at the injection site, known allergy to local anesthetics, long-term steroid therapy, use of premedications that could affect outcomes (opioids, ketamine, NSAIDs), diabetes mellitus, and hepatic or renal disease.

### Sample size

Sample size was calculated to detect differences in the primary outcome (duration of analgesia, measured in hours). Variance estimates were derived from Pawan P.B, (2019), which reported mean durations of 7.5 ± 1.0 hours in controls and 17.3 ± 3.7 hours with dexamethasone. Using α = 0.05 and power = 90%, the minimum required sample size was 7 per group. To improve precision, external validity, and account for potential attrition, we enrolled 30 participants per group. A sensitivity analysis was performed using variance estimates ±20% of those reported in the reference study. These calculations confirmed that a sample size of 30 per group remained sufficient to detect the observed effect.

### Research variables

Primary Outcome: Duration of postoperative analgesia, was defined as the time from block completion to the first request for rescue analgesia or Numeric Rating Scale (NRS) ≥ 4. Analgesic administration thresholds were standardized across participants.

Secondary Outcome: Onset of sensory and motor block, Postoperative pain scores, Analgesic consumption

Independent variables: Perineural Bupivacaine, intravenous dexamethasone injection, intravenous normal saline

Patient variables: Socio-demographic & clinical characteristics.

### Data collection and anesthesia protocol

Sociodemographic and clinical data (age, sex, education, religion, ethnicity, weight, height, ASA status, BMI, prior anesthesia/surgery history, analgesic doses and timing) were collected using a structured checklist. A standard pre-anesthetic assessment was performed, and informed consent obtained. The data collection started on September 23, 2024 and ended on January 17, 2025.

All SCBPBs were performed by anesthetists with at least two years of experience in ultrasound-guided techniques. A designated anesthetist administered the block independently. The patients were placed in a supine position. Secure and functional intravenous line was opened. Vital signs were monitored for blood pressure, pulse, and SpO2 and were placed in the arms as required. The site of SCBPB was identified before the drug was administered. A thorough asceptic techniques was used during the block. The brachial nerve plexus was identified using ultrasound by applying an in-plane technique. After drug administration, vital signs were recorded, and sensory/motor block onset was assessed every 2 minutes for 15 minutes. Sensory block assessed via pinprick on a 3-point scale (2 = normal sensation, 1 = loss of pinprick sensation, 0 = loss of light touch). Motor block assessed using the modified Bromage scale (2 = complete block, 1 = reduced strength, 0 = normal function). Vital signs were monitored every 10 minutes until the end of surgery. No single patient in the study group was given sedation.

Postoperative pain intensity, time to first analgesic request, type and total analgesic use within 24 hours were recorded. Patients were instructed to self-report pain using the Numeric Rating Scale (NRS), a 10 cm line where 0 = no pain and 10 = worst imaginable pain. Pain scores were recorded at 1, 2, 4, 6, 8, 12, and 24 hours postoperatively. Adverse events (nausea, vomiting, seizures, hypotension, bradycardia, respiratory depression, allergic reactions) were documented. There were no any adverse events throughout the study, details explained in table form in the result section below. Data completeness and consistency were verified.

### Randomization and blinding

Participants were randomized after eligibility confirmation and consent, immediately prior to block administration. The random sequence was generated using a simple lottery method. Sealed opaque envelopes containing group assignments were prepared by an independent staff member not involved in patient care. Allocation concealment was ensured until the envelope was opened at the time of intervention. Block randomization or stratification was not used.

### Intervention

Ethical approval was obtained from the Orotta College of Medicine and Health Sciences Ethics Committee and the Ministry of Health’s Department of Research and Human Resources. The study adhered to CONSORT guidelines. Additional permission was granted by the hospital administration. Participants were randomly assigned to two groups using a simple lottery method. Experimental group received 20 ml of 0.5% bupivacaine plus 8 mg IV dexamethasone and Control group received 20 ml of 0.5% bupivacaine with 2 ml of normal saline.

Postoperative monitoring and pain assessments were conducted in the ward at seven time points (1st, 2nd, 4th, 6th, 8th, 12th, and 24th hours). NRS scores and checklist data were used to evaluate pain. Patients requiring additional analgesia or experiencing complications were managed accordingly.

### Trial registration

The trial was registered retrospectively in the Pan African Clinical Trial Registry (PACTR202509579633417). Retrospective registration occurred due to administrative delays and a shortage of stable internet services, which made it difficult to access the registry platform and we acknowledge the risk of reporting bias. Ethical approval was obtained prior to recruitment, and the study protocol was followed throughout. We acknowledge retrospective registration as a limitation.

### Data analysis

data were entered into SPSS version 26.0 (IBM Corp., Chicago, USA). After cleaning, descriptive and inferential statistics were performed. Continuous variables (e.g., duration of analgesia, onset times) were compared using independent samples t‑tests when normally distributed. Mann–Whitney U tests and Shapiro-Wilk and Levene’s test were performed to confirm normality and homogeneity of variance prior to parametric analysis. Categorical variables (e.g., analgesic use, adverse events) were compared using chi‑square or Fisher’s exact tests. Pain scores across time points were analysed using repeated‑measures ANOVA to account for multiple comparisons. Regression analyses adjusting for baseline imbalances (BMI, religion, ethnicity) were performed, with effect sizes and 95% confidence intervals reported.

### Generalizability

This study supports the use of dexamethasone as an adjuvant in low-resource settings, offering prolonged analgesia and reduced burden on healthcare systems.

### Trial status

Recruitment and data collection are complete.

## Results

**Fig 1:**
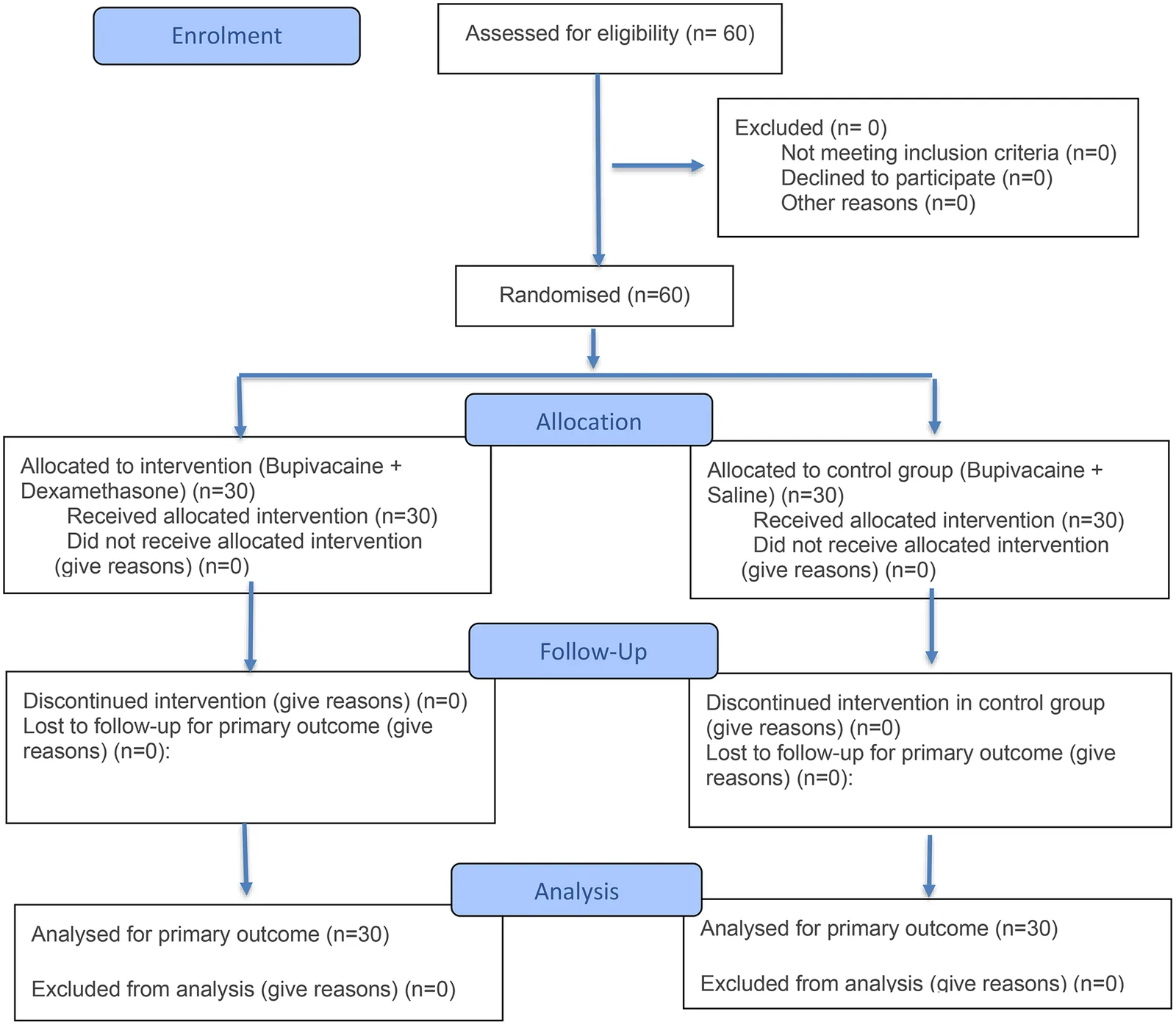
CONSORT Flowchart of the participants. Flow diagram of the progress through the phases of a randomised trial of two groups (that is, enrolment, intervention allocation, follow-up, and data analysis). Citation: Hopewell S, Chan AW, Collins GS, Hróbjartsson A, Moher D, Schulz KF, et al. CONSORT 2025 Statement: updated guideline for reporting randomised trials. BMJ. 2025; 388:e081123. https://dx.doi.org/10.1136/bmj-2024-081123. © 2025 Hopewell et al. This is an Open Access article distributed under the terms of the Creative Commons Attribution License (https://creativecommons.org/licenses/by/4.0/), which permits unrestricted use, distribution, and reproduction in any medium, provided the original work is properly cited.

Sixty patients were assessed for eligibility between September 2024 and January 2025. All 60 met inclusion criteria and were randomized into two groups (30 per group). No patients were lost to follow-up, and no outcome data were missing. The flow of participants through each stage of the trial is shown in Fig 1.

### Baseline characteristics of the patients

The baseline demographic and clinical characteristics of the patients are summarized in Table 1. A total of 60 patients were enrolled, with 30 in each group. The median age was comparable between groups (p = 0.317). Female participants comprised 26.7% in both groups. All patients were ASA I, and only one patient in the experimental group had a previous history of anesthesia.

**Table 1:** Baseline characteristics of the patients.

Variable	Control (n = 30)	Experimental (n = 30)	P value
Age (years, mean ±SD)	31.3 ± 22	30.3 ± 27	0.317
Sex, n (%)	Male: 22 (73.3%) Female: 8 (26.7%)	Male: 22 (73.3%) Female: 8 (26.7%)	1.000
BMI (kg/m^2^, mean ± SD)	20.21 ± 2.14	19.21 ± 1.47	0.039
ASA status, n (%)	ASA I: 30 (100%)	ASA I: 30 (100%)	–
Previous Anesthesia, n (%)	Yes: 0 (0%) No: 30 (100%)	Yes: 1 (3.3%) No: 29 (96.7%)	1.000
Heart rate (beats/min, mean ± SD)	78.6 ± 10.4	81.8 ± 8.9	0.204
Systolic BP (mmHg, mean ± SD)	118.2 ± 8.6	118.8 ± 8.1	0.782
Diastolic BP (mmHg, mean ± SD)	74.1 ± 8.3	76.5 ± 7.4	0.228
Respiratory rate (breaths/min, mean ± SD)	14.1 ± 1.9	14.0 ± 1.5	0.817
SpO_2_ (% mean ± SD)	98.7 ± 1.1	98.6 ± 0.9	0.702

Baseline characteristics of patients in the control and experimental groups. Values are presented as mean ± SD or n (%). P values were calculated using independent samples t-test for continuous variables and Fisher’s exact test for categorical variables. ASA = American Society of Anesthesiologists; BMI = Body Mass Index; BP = Blood Pressure; SpO_2_ = Oxygen saturation.

BMI was significantly lower in the experimental group compared to the control group (p = 0.039). No significant differences were observed in baseline vital signs, including heart rate, systolic and diastolic blood pressure, respiratory rate, and oxygen saturation (all p > 0.2). These findings confirm that the groups were generally comparable at baseline, except for BMI.

### Intraoperative vital signs

**Fig 2:**
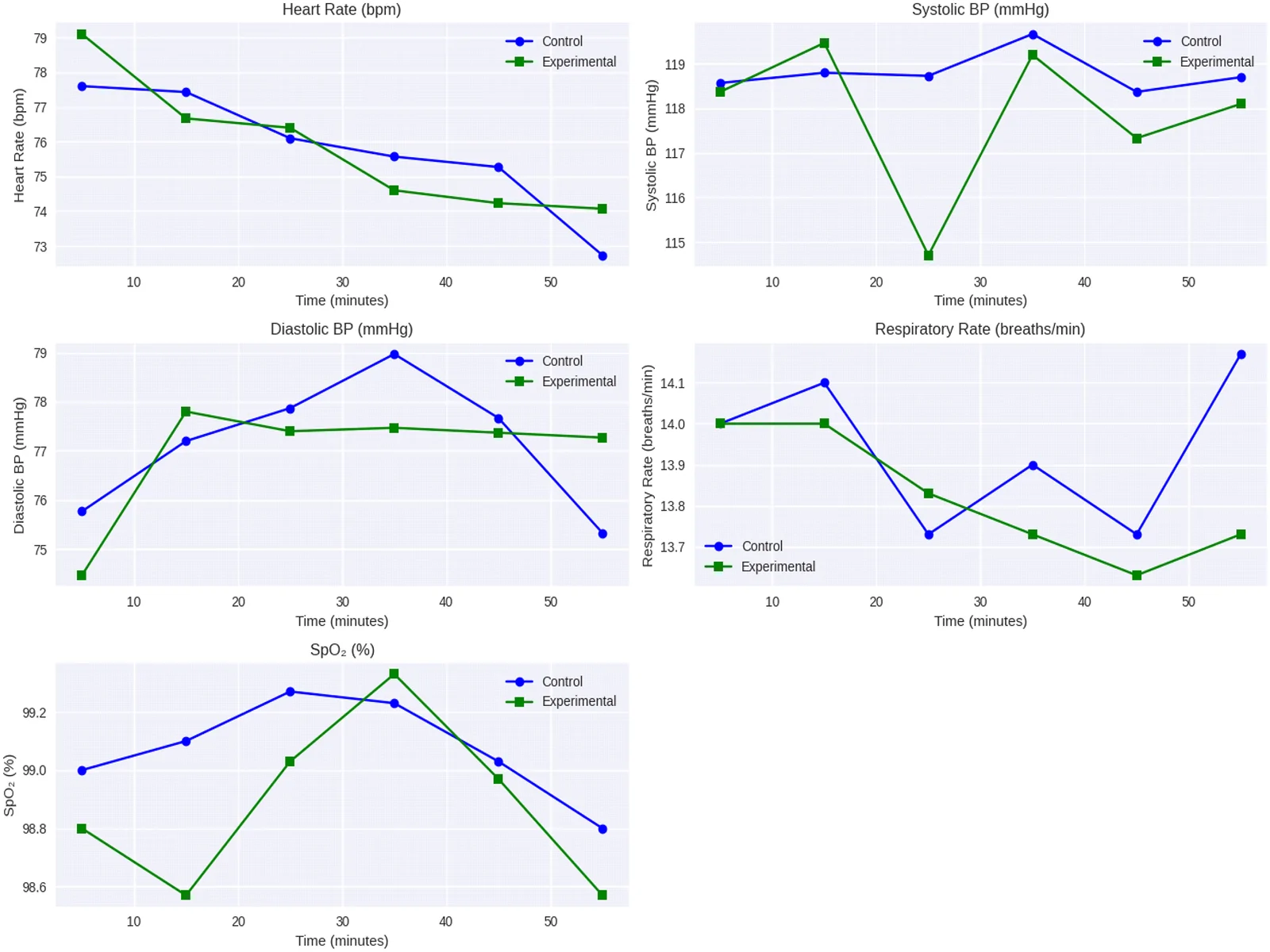
Intraoperative vital signs in control and experimental groups. Mean values (± SD) for heart rate (A), systolic blood pressure (B), diastolic blood pressure (C), respiratory rate (D), and SpO_2_ (E) are plotted at 5, 15, 25, 35, 45, and 55 minutes intraoperatively. No significant differences were observed between groups except for SpO_2_ at 15 minutes (p = 0.021).

Intraoperative monitoring showed no significant differences in heart rate, systolic or diastolic blood pressure, or respiratory rate between groups at any recorded time point. Oxygen saturation was slightly higher in the control group at 15 minutes (p = 0.021). These trends are illustrated in Fig 2.

Regression analyses adjusting for baseline imbalances in BMI, religion, and ethnicity are presented in S1 Table. These analyses confirmed that the observed differences in analgesia duration remained significant after adjustment (β = 9.62 hours, 95% CI 8.41–10.83, p < 0.001). None of the covariates materially altered the primary outcome.

### Block characteristics and analgesia duration

The duration of surgery was similar between groups. However, the duration of analgesia was significantly longer in the experimental group compared to the control group (p < 0.001). The onset of sensory and motor block was also faster in the experimental group. These findings are summarized in Table 2.

**Table 2:** Block characteristics and analgesia duration.

Variable	Control	Experimental	P - value
	Mean ± SD	Mean ± SD	
Duration of surgery, in minutes	64.60 ± 14.10	67.33 ± 16.75	0.497
Duration of analgesia, in hours	7.57 ± 1.04	17.36 ± 3.75	**<0.001**
Onset of sensory block, in minutes	12.03 ± 2.39	6.53 ± 1.04	**<0.001**
Onset of motor block, in minutes	15.37 ± 1.96	9.63 ± 1.38	**<0.001**

Values are presented as mean ± SD. P values were calculated using independent samples t-test for continuous variables. SD: Standard Deviation.

### Postoperative pain scores

**Fig 3:**
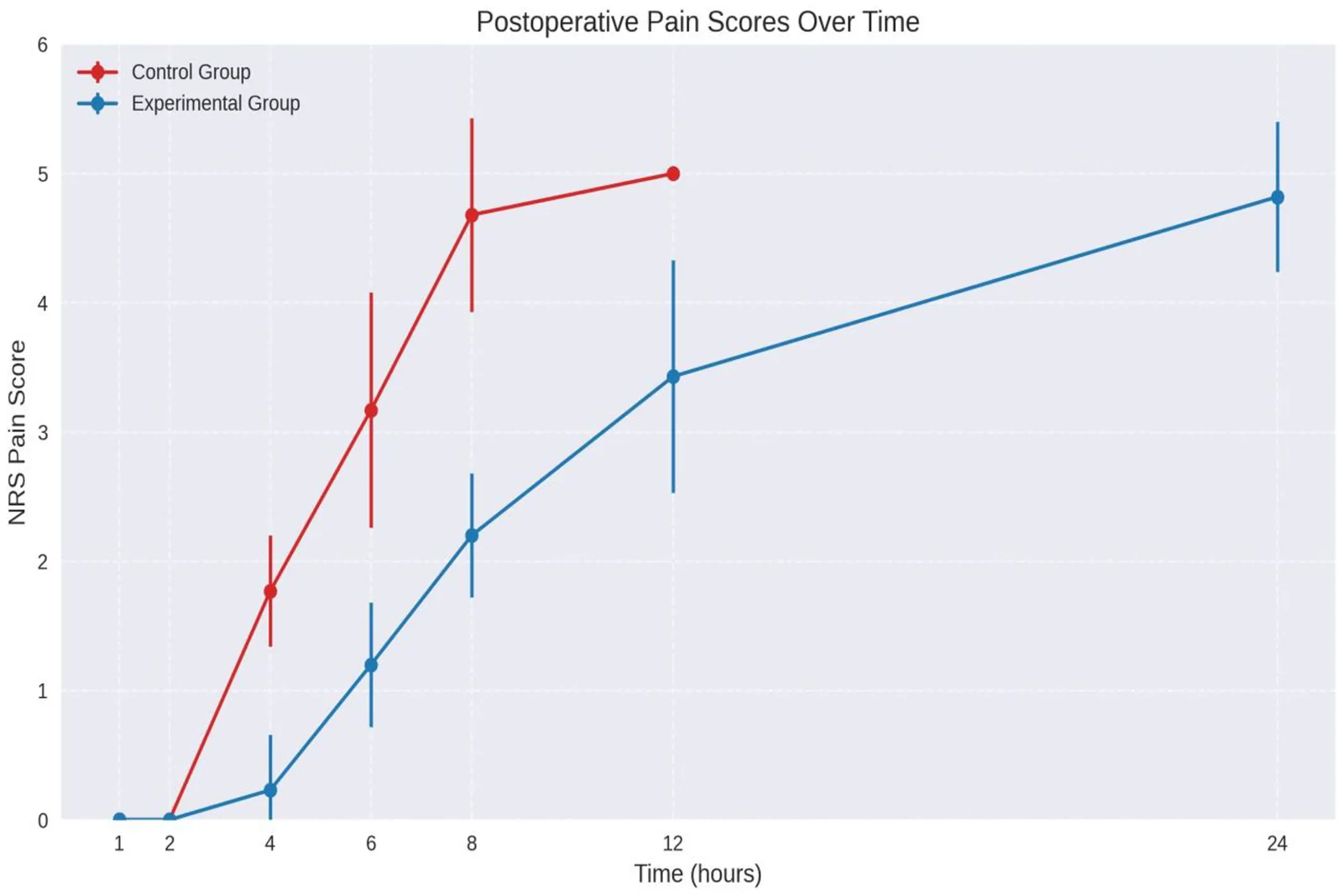
Postoperative pain scores in control and experimental groups. Mean NRS pain scores (± SD) are plotted at 1, 2, 4, 6, 8, 12, and 24 hours postoperatively. The experimental group (bupivacaine + IV dexamethasone) consistently demonstrated lower pain scores compared to the control group (bupivacaine alone), with statistically significant differences at 4, 6, 8, and 12 hours (p < 0.001). At 24 hours, pain scores were descriptively lower in the experimental group, but statistical comparison was not performed due to widespread rescue analgesia.

Postoperative pain intensity was assessed using the Numeric Rating Scale (NRS) at seven time points. No differences were observed at 1 and 2 hours. However, pain scores were significantly lower in the experimental group at 4, 6, 8, and 12 hours (all p < 0.001). At 24 hours, pain scores remained lower in the experimental group, though not statistically tested due to widespread rescue analgesia. These trends are illustrated in Fig 3.

### Analgesic consumption

Diclofenac use was significantly higher in the control group compared to the experimental group (p < 0.001). Ibuprofen use was not significantly different between groups, while paracetamol use was equal in both groups. These findings are summarized in Table 3.

**Table 3:** Analgesic consumption in the control and experimental groups.

Analgesic	Control (n = 30)	Experimental (n = 30)	P value
Diclofenac, n (%)	30 (100%)	14 (46.7%)	≤ 0.001
Ibuprofen, n (%)	7 (23.3%)	3 (10.0%)	0.299
Paracetamol, n (%)	10 (33.3%)	10 (33.3%)	1.000

Values are presented as n (%). P values calculated using Fisher’s exact test.

Post‑hoc power analysis indicated >80% power for detecting differences in onset times and analgesic consumption, supporting the robustness of these findings. Secondary outcomes were exploratory and interpreted with caution.

### Adverse events

No adverse events were observed in either group. Monitored complications included nausea, vomiting, hypotension, bradycardia, respiratory depression, allergic reactions, and seizures. These findings are summarized in Table 4.

**Table 4:** Adverse events in the control and experimental groups.

Adverse Events	Control (n = 30)	Experimental (n = 30)
Nausea	0	0
Vomiting	0	0
Hypotension	0	0
Bradycardia	0	0
Allergic Reactions	0	0
Respiratory depression	0	0
Seizures	0	0

Values are presented as n (%). No adverse events were observed in either group during the perioperative period.

## Discussion

This randomized clinical trial demonstrated that the addition of 8 mg intravenous dexamethasone to 0.5% bupivacaine in supraclavicular brachial plexus block significantly prolonged postoperative analgesia, accelerated the onset of sensory and motor block, and reduced postoperative analgesic consumption compared with bupivacaine alone. Pain scores were consistently lower in the dexamethasone group at 4, 6, 8, and 12 hours, supporting our hypothesis that systemic corticosteroid administration enhances block quality and duration.

Our findings are consistent with earlier reports showing that dexamethasone prolongs the duration of peripheral nerve blocks [8,10,14]. Pawan et al. [15] reported mean analgesia durations of 17.3 hours with dexamethasone compared to 7.5 hours in controls, closely mirroring our results. Other studies have highlighted both perineural and intravenous routes as effective, though the perineural route has raised concerns about neurotoxicity [16]. The intravenous route, as used here, offers a safer and more practical alternative, particularly in resource limited settings where ultrasound guidance and sterile catheter techniques may not be consistently available. Our results therefore add to the growing evidence that intravenous dexamethasone is a viable adjunct for prolonging brachial plexus block analgesia.

The magnitude of effect observed in this study was substantial, with a mean difference of nearly 10 hours in analgesia duration between groups. Faster onset of sensory and motor block in the dexamethasone group may be explained by its anti-inflammatory properties and modulation of nociceptive transmission [11–13,17,18]. The reduction in diclofenac use further supports the clinical relevance of this intervention, as decreased reliance on rescue analgesics can improve patient comfort and reduce side effects. Importantly, no adverse events were reported, reinforcing the safety profile of intravenous dexamethasone [19,20]. However, the absence of complications should be interpreted cautiously given the modest sample size [21,22].

Finally, we acknowledge that all enrolled patients were ASA I, representing a healthy, low‑risk cohort. While this enhances internal validity by reducing confounding from comorbidities, it limits generalizability to broader perioperative populations, including diabetic, immunocompromised, or higher‑risk patients in whom dexamethasone’s risk–benefit profile may differ. Future studies should therefore include patients with diverse ASA classifications to better assess external validity.

### Strengths and limitations

Strengths of this study include its randomized design, standardized anesthesia protocol, and comprehensive pain assessment at multiple postoperative time points. The operational definition of analgesia duration was clear and consistent, and outcome assessors were trained anesthetists, reducing measurement bias. Figures were used to present vital signs and pain scores, minimizing redundancy and improving clarity.

Several limitations must be acknowledged. First, the trial was registered retrospectively, which may raise concerns about protocol transparency and risk of reporting bias. Second, although randomization was performed using sealed envelopes, baseline imbalances in BMI, religion, and ethnicity were observed; regression analysis was used to adjust for these differences. Third, blinding was partial, as anesthetists administering the block were aware of group allocation and although ward nurses recording NRS scores were not aware of group allocation, they were aware that patients were enrolled in a clinical study. This partial blinding may have introduced expectation bias, as awareness of participation can influence observer diligence and patient reporting. Future studies should ensure full blinding of outcome assessors to minimize this risk. Fourth, although the sample size calculation was based on prior variance estimates, sensitivity analyses confirmed its adequacy, and post‑hoc power commentary supports the robustness of secondary findings. Nonetheless, secondary outcomes remain exploratory and should be interpreted cautiously. Fifth, the study was conducted at a single center with a relatively small sample size, limiting generalizability. Finally, pain scores at 24 hours were not statistically analyzed due to widespread rescue analgesia use, which may have introduced bias. We therefore analyzed pain scores at standardized intervals using repeated‑measures ANOVA and t‑tests, which allowed consistent comparison. Future studies with larger sample sizes and longer follow‑up could incorporate Kaplan–Meier survival analysis to strengthen external validity.

Despite these limitations, the results suggest that intravenous dexamethasone is a safe, effective, and practical adjunct to bupivacaine in supraclavicular brachial plexus block [23,24]. Its low cost, ease of administration, and favorable safety profile make it particularly attractive in low resource settings such as Eritrea. Larger multicenter trials are warranted to confirm these findings, explore optimal dosing, and assess long term outcomes. Future research should also investigate patient reported quality of recovery and functional outcomes, which were beyond the scope of this study.

## Conclusion

In summary, intravenous dexamethasone significantly prolonged postoperative analgesia, accelerated block onset, and reduced analgesic consumption without adverse events. These findings support its use as a practical adjunct to bupivacaine in supraclavicular brachial plexus block, with potential to improve postoperative pain management in resource limited environments.

## Supporting information

S1 Table Supplementary table providing extended data analyses and additional results supporting the main findings of the trial. (DOCX)

S1 Protocol Complete study protocol document outlining methodology, inclusion/exclusion criteria, anesthesia procedures, and ethical approvals. (DOCX)

S1 Checklist CONSORT 2025 checklist confirming adherence to randomized clinical trial reporting standards. (DOCX)
