# Supplementary material for: The effect of intravenous dexamethasone in supraclavicular brachial plexus block at Halibet National Referral Hospital, Eritrea 2024–2025: A randomized clinical trial
Source: PLoS One. 2026 Sep 3;21(9):e0338067. doi: 10.1371/journal.pone.0338067 (PMC13541117; doi:10.1371/journal.pone.0338067)
Supplement: Supplementary file 1 [file pone.0338067.s001.docx]

**Supplementary Table 1. Regression analyses adjusted for baseline imbalances (BMI, Religion, Ethnicity)**

| **Variable** | **Coefficient (β)** | **Adjusted Effect Estimate (hours)** | **95% Confidence Interval** | **P value** |
| --- | --- | --- | --- | --- |
| **Dexamethasone vs. Control** | 9.62 | 17.36 vs. 7.57 | 8.41 – 10.83 | <0.001 |
| **BMI (per unit increase)** | –0.12 | — | –0.25 – 0.01 | 0.07 |
| **Religion (Christian vs. Muslim)** | 0.45 | — | –0.38 – 1.28 | 0.28 |
| **Ethnicity (Group A vs. B)** | 0.63 | — | –0.22 – 1.48 | 0.14 |

Linear regression model adjusting for baseline imbalances in BMI, religion, and ethnicity.

Primary outcome: duration of postoperative analgesia (hours).

Effect estimates for dexamethasone remained significant after adjustment.

Non‑significant covariates are reported for transparency.
