# Supplementary material for: The effect of intravenous dexamethasone in supraclavicular brachial plexus block at Halibet National Referral Hospital, Eritrea 2024–2025: A randomized clinical trial
Source: PLoS One. 2026 Sep 3;21(9):e0338067. doi: 10.1371/journal.pone.0338067 (PMC13541117; doi:10.1371/journal.pone.0338067)
Supplement: Supplementary file 2 [file pone.0338067.s002.docx]

Study Protocol

Title: Effect of Intravenous Dexamethasone in Prolonging Analgesia After Supraclavicular Brachial Plexus Block in Upper Limb Surgeries at Halibet National Referral Hospital, Asmara, Eritrea, 2024–2025

Authors

Michael Beraki Mengistu*, Senay Amare Habtu, Abel Mussie Elias, Sabrina Hashim Idris, Kisanet Huruy Abraha *Corresponding author: Mikoberaki88@gmail.com Institution: Orotta College of Medicine and Health Sciences

Abstract

Background: Postoperative pain following upper limb surgeries can significantly affect recovery and healthcare costs. Supraclavicular brachial plexus block provides effective regional anesthesia, but its duration is limited. Dexamethasone, a glucocorticoid, may enhance and prolong analgesia when used as an adjunct.

Objective: To evaluate the effect of intravenous dexamethasone on the duration of postoperative analgesia following supraclavicular brachial plexus block with bupivacaine.

Methods: A randomized, double-blind, controlled clinical trial will be conducted at Halibet National Referral Hospital, Eritrea, involving 60 patients undergoing upper limb surgery.

Group A: 0.5% bupivacaine (20 ml) + 2 ml normal saline

Group B: 0.5% bupivacaine (20 ml) + 2 ml (8 mg) dexamethasone

Pain scores, time to first rescue analgesia, and total analgesic consumption will be recorded. Data will be analyzed using SPSS v26, with statistical significance set at p < 0.05.

Expected Outcomes: Intravenous dexamethasone is anticipated to significantly prolong analgesia duration, reduce analgesic consumption, and accelerate the onset of sensory and motor block.

Trial Registration: The study will be registered in the Pan African Clinical Trial Registry.

Introduction

Pain is a multidimensional experience, defined by the International Association for the Study of Pain (IASP) as “an unpleasant sensory and emotional experience associated with actual or potential tissue damage” [1]. Postoperative pain (POP) remains undertreated, leading to delayed recovery, increased healthcare costs, and risk of chronic pain due to central sensitization [2][3].

Regional anesthesia, particularly brachial plexus block (BPB), is widely used for upper limb surgeries. However, the duration of analgesia is limited by the pharmacokinetics of local anesthetics [4][5]. Adjuvants such as opioids, clonidine, dexmedetomidine, and corticosteroids have been explored to prolong block duration [6][8].

Dexamethasone, a long-acting glucocorticoid (half-life >36 hours), prolongs nerve block duration by inhibiting nociceptive C-fiber transmission, modulating potassium channel activity, and inducing vasoconstriction [10–12]. Previous studies have shown that intravenous dexamethasone enhances block duration and reduces postoperative pain intensity without major adverse effects [13–16].

No studies have yet investigated this approach in Eritrea, making this trial both novel and clinically relevant.

Objectives

General Objective: To evaluate the effect of intravenous dexamethasone on the duration of postoperative analgesia following supraclavicular brachial plexus block with bupivacaine.

Specific Objectives:

To compare the duration of postoperative analgesia between the two groups.

To compare the onset of sensory block between the groups.

To compare the onset of motor block between the groups.

To determine postoperative analgesic consumption in both groups.

Methods

Study Design: Randomized, double-blind, controlled clinical trial.

Study Setting: Halibet National Referral Hospital, Asmara, Eritrea.

Study Period: January 2024 – December 2025.

Participants:

Inclusion criteria: Adults aged 18–65 years undergoing elective upper limb surgery under supraclavicular brachial plexus block.

Exclusion criteria: Patients with contraindications to regional anesthesia, allergy to study drugs, chronic opioid use, or systemic corticosteroid therapy.

Sample Size: 60 patients (30 per group).

Randomization & Blinding: Participants will be randomly assigned to Group A or Group B using computer-generated random numbers. Both patients and outcome assessors will be blinded to group allocation.

Interventions:

Group A: 0.5% bupivacaine (20 ml) + 2 ml normal saline.

Group B: 0.5% bupivacaine (20 ml) + 2 ml (8 mg) intravenous dexamethasone.

Outcome Measures:

Primary outcome: Duration of postoperative analgesia (time to first rescue analgesia).

Secondary outcomes: Pain scores (VAS), onset of sensory and motor block, total postoperative analgesic consumption.

Data Collection & Analysis: Pain scores will be recorded at regular intervals postoperatively. Data will be analyzed using SPSS v26. Continuous variables will be compared using t-tests, categorical variables with chi-square tests. p < 0.05 will be considered statistically significant.

Ethical Considerations: Approval will be obtained from the Orotta College of Medicine and Health Sciences Ethics Committee. Written informed consent will be secured from all participants.

**References**

Institute of Medicine. Relieving Pain in America: A Blueprint for Transforming Prevention, Care, Education, and Research. Washington (DC): National Academies Press; 2011.

Brennan F, Carr DB, Cousins M. Pain management: a fundamental human right. Anesth Analg. 2007;105(1):205‑21.

Desmet M, Braems H, Reynvoet M, et al. IV and peri‑neural dexamethasone are equivalent in increasing the analgesic duration of single‑shot interscalene block with ropivacaine for shoulder surgery. Acta Anaesthesiol Belg. 2013;64(2):57‑63.

Adhikary SD, Armstrong K, Chin KJ. Perineural entrapment of an interscalene stimulating catheter. Anaesth Intensive Care. 2012;40(1):166‑8.

Nwosu ADG, Chukwudi L, Onwuasoigwe O. Redefining the role of analgesic adjuvants in pain management. Niger J Clin Pract. 2023;26(4):543‑9.

Choi S, Rodseth R, McCartney CJ. Effects of dexamethasone as a local anaesthetic adjuvant for brachial plexus block. Reg Anesth Pain Med. 2014;39(6):579‑88.

Edinoff AN, Hasoon G, Patil S, et al. Adjuvant drugs for peripheral nerve blocks: The role of alpha‑2 agonists, dexamethasone, midazolam, and NSAIDs. J Pain Res. 2021;14:2855‑69.

Hewson D, Borton N, McCartney C, Hardman J. Dexamethasone and peripheral nerve blocks. Anaesthesia. 2019;74(5):661‑70.

Waldron NH, Jones CA, Gan TJ, et al. Impact of perioperative dexamethasone on postoperative analgesia and side effects. Anesth Analg. 2013;117(1):206‑17.

Oliveira J. Does the addition of dexamethasone to local anesthetic used for peripheral nerve block prolong analgesia in the surgical patient? Pain Physician. 2015;18(1):E1‑12.

Suzuki S, Gerner P, Lirk P. Local anesthetics. In: Evers AS, Maze M, editors. Pharmacology and Physiology for Anesthesia. Philadelphia: Elsevier; 2019. p. 331‑48.

Saba S, Babar A. The effect of intravenous dexamethasone on prolonging analgesia after supraclavicular brachial plexus block. J Anesth Res Sci Stud. 2020;8(2):45‑51.

Dar FA, Najar MS, Jan N. Effect of addition of dexamethasone to ropivacaine in supraclavicular brachial plexus block. Indian J Pain. 2013;27(3):129‑33.

Alarasan AK, Arumugam J, Choudhary B, et al. Effect of dexamethasone in low volume supraclavicular brachial plexus block. J Anaesthesiol Clin Pharmacol. 2016;32(3):387‑91.

El‑Baradey GF, El‑Naby M. The efficacy of adding dexamethasone or epinephrine to 0.5% bupivacaine in supraclavicular brachial plexus block. Saudi J Anaesth. 2014;8(4):507‑13.

Abdallah FW, Johnson J, Chan V, et al. Intravenous dexamethasone and perineural dexamethasone similarly prolong the duration of analgesia after supraclavicular brachial plexus block. Reg Anesth Pain Med. 2015;40(2):125‑32.

El Azzazi HM, El Azzazi AA, Mohamed MM. Effect of adding dexamethasone to bupivacaine in ultrasound‑guided supraclavicular brachial plexus block versus bupivacaine alone for upper limb orthopedic surgery. Egypt J Hosp Med. 2018;70(7):1152‑8.

Baral PP, Pokhrel L. A study of dexamethasone as an adjuvant with bupivacaine for supraclavicular brachial plexus block. IOSR J Dent Med Sci. 2019;18(3):72‑6.

Al Maruf HN. Dexamethasone added to bupivacaine prolongs the duration of analgesia for supraclavicular brachial plexus block. J Anesth Clin Res. 2018;9(5):100‑5.

Kumar B, Kumari S. Effectiveness of dexamethasone as an adjuvant to bupivacaine in supraclavicular brachial plexus block. Dinkum. 2024;12(1):33‑9.

Islam SM, Hossain M, Maruf AA. Effect of addition of dexamethasone to local anesthetic in supraclavicular brachial plexus block. J Armed Forces Med Coll Bangladesh. 2011;7(1):15‑20.

De Oliveira GS, Almeida MD, Benzon HT, McCarthy RJ. Perioperative single‑dose systemic dexamethasone for postoperative pain. Anesthesiology. 2011;115(3):575‑88.

Yadav RK, Singh B, Kumar P, Singh SN. Effectiveness of addition of neostigmine or dexamethasone to local anesthetic in providing perioperative analgesia for brachial plexus block. J Anaesth Clin Pharmacol. 2008;24(1):7‑10.
