# Supplementary material for: The effect of intravenous dexamethasone in supraclavicular brachial plexus block at Halibet National Referral Hospital, Eritrea 2024–2025: A randomized clinical trial
Source: PLoS One. 2026 Sep 3;21(9):e0338067. doi: 10.1371/journal.pone.0338067 (PMC13541117; doi:10.1371/journal.pone.0338067)
Supplement: Supplementary file 9 [file pone.0338067.s009.docx]

**August 3, 2026**

**Response to Reviewers**

We thank the Academic Editor and Reviewers for their continued constructive feedback. We have carefully revised the manuscript to address each point raised. Below, we provide point‑by‑point responses.

**Reviewer #1**

- We thank the reviewer for constructive and thoughtful comments, which have helped improve the clarity and structure of the manuscript.

**Reviewer #7**

- We appreciate the reviewer’s comments. All recommendations raised in the previous cycle have been incorporated, and the manuscript has been structured and clarified to address the scientific inquiries.

**Reviewer #8**

- We thank the reviewer for valuable suggestions. The abstract has been revised according to the recommendations, improving clarity and alignment with the study’s main objectives.

**Closing**

We believe these revisions address all outstanding concerns from Reviewers #1, #7, and #8. The manuscript has been extensively revised and polished to meet the criteria of PLOS ONE. Given the multiple independent reviews already completed, we respectfully request that this version be considered suitable for publication.
