# Supplementary material for: The effect of intravenous dexamethasone in supraclavicular brachial plexus block at Halibet National Referral Hospital, Eritrea 2024–2025: A randomized clinical trial
Source: PLoS One. 2026 Sep 3;21(9):e0338067. doi: 10.1371/journal.pone.0338067 (PMC13541117; doi:10.1371/journal.pone.0338067)
Supplement: Supplementary file 10 [file pone.0338067.s004.docx]

In this study, authors investigated the effect of intravenous dexamethasone combined with bupivacaine on prolonging postoperative analgesia following supraclavicular brachial plexus block. They reported that the addition of 8 mg intravenous dexamethasone to 0.5% bupivacaine in supraclavicular brachial plexus blocks significantly prolonged postoperative analgesia, accelerated the onset of sensory and motor block, and reduced analgesic consumption.

**The following major revisions are necessary for this study:**

-What is the authors' hypothesis? On what basis did they conclude that intravenous dexamethasone might improve the success of the block? The hypothesis should be written at the end of the introduction, and the argument should be written based on that hypothesis.

- The randomization process should be described in detail, specifying at what stage the patients were randomized. Clarify how the random sequence was generated.Specify who prepared the sealed opaque envelopes and how allocation concealment was ensured. Explicitly state whether block randomization or stratification was considered.

**-**The trial was registered retrospectively, which is a major limitation for a randomized clinical trial. The authors should: provide a clear justification for retrospective registration. Explicitly acknowledge retrospective registration as a limitation.

-There's a problem with calculating the sample size. Getting 7 is unlikely. It needs to be recalculated. The sample size calculation suggests that only 7 participants per group were required, yet 30 were included in each group. While over-recruitment can be acceptable, the rationale for this decision is not adequately justified. Additionally: The reported means (μ₁ and μ₂) appear unusually large and their units are unclear. The reference study used for variance estimation should be clearly cited and explained.

-Outcome Definitions and Measurement Consistency: The definition of “duration of analgesia” should be clarified (e.g., time from block completion to first request for rescue analgesia). Pain at 24 hours is reported descriptively but not statistically compared; the rationale for this omission should be explained. The use of NRS is appropriate, but the manuscript alternates between NRS and VAS terminology, which should be standardized.

-Standard statistical analysis statements should not be written; it should be clearly stated which parameter was compared with which test.

-Many grammatical errors and awkward phrasings are present (e.g., “the effective of dexamethasone” instead of “the effect of dexamethasone”). Professional language editing is strongly recommended.

**-**Clarify abbreviations at first mention and avoid redundancy.

**-**Figures should include clear legends and units. Figure numbering appears inconsistent (e.g., Figure 1 vs. Figure 2 in the text). Consort flow diagram??

-It is unnecessary to present the same data in both figures and tables.

- Parameters like ‘Educational level, Religious affiliation, Ethnicity’ are unnecessary as they do not affect the study results. The total column in Tables 1 and 2 is also unnecessary. Data can be presented in a single row; simply stating male/female or the number of individuals with an anesthesia history would suffice. The tables should be reorganized, and Tables 1 and 2 should be merged.

-Table 3's RR and spo2 are unnecessary. Changes in blood pressure should be presented graphwise; a table is not needed. Table 6 should be rearranged, and Table 5 and Figure 3 should be shown only as figures, with data written below the figure. Figure 1?

-The discussion section should include figures and mean-standard deviations. The discussion should be a section interpreting the results. The discussion should be revised to include the results of studies in the literature and the factors influencing the differences in results. The new contribution of this study to the literature should be stated.

-The discussion occasionally overstates causality given the study limitations. Comparisons with existing literature are appropriate but could be more concise. The novelty of intravenous (rather than perineural) dexamethasone in this specific setting should be more clearly emphasized.

In conclusion, major revisions are necessary.

Sincerely yours…
