# Supplementary material for: The effect of intravenous dexamethasone in supraclavicular brachial plexus block at Halibet National Referral Hospital, Eritrea 2024–2025: A randomized clinical trial
Source: PLoS One. 2026 Sep 3;21(9):e0338067. doi: 10.1371/journal.pone.0338067 (PMC13541117; doi:10.1371/journal.pone.0338067)
Supplement: Supplementary file 11 [file pone.0338067.s005.docx]

**Response to reviewers**

We thank the Academic Editor and Reviewers for their constructive comments. We have revised the manuscript accordingly. Below, we provide point‑by‑point responses.

**Reviewer #1 (Statistical Reviewer)**

**1. Power calculations and primary outcome definition**

**Response**: We appreciate this important observation. We now define the primary outcome (duration of postoperative analgesia) before presenting the power calculation. We added the reference study used for variance estimates and clarified that μ₁ and μ₂ represent mean duration in hours. We acknowledge the large assumed effect size and discuss this limitation in the Methods and Discussion.

**2. Non‑inferiority design**

**Response**: We acknowledge that our design resembles a non‑inferiority comparison. We added a paragraph in the Discussion clarifying this limitation and framed conclusions more cautiously.

**3. Writing quality**

**Response**: We thank the reviewer for this candid feedback. The manuscript has been thoroughly edited to improve flow, reduce repetition, and merge short paragraphs.

**4. Missing data**

**Response**: We confirm that all data were complete; no participants were lost to follow‑up. This is now explicitly stated in the first part of the result section.

**5–6. Baseline imbalances and regression analysis**

**Response**: We accept the reviewer’s concern. We acknowledge imbalances in religion, ethnicity, and BMI. We performed regression analyses adjusting for these variables and report effect sizes with confidence intervals. Results are presented in the revised Results section.

**7. Retrospective registration**

**Response**: We acknowledge this limitation and explain that registration was delayed due to administrative issues and instable internet services. This is now discussed in the Methods and Discussion.

**Minor: Outcome assessors**

**Response**: Thank you for pointing this out. Outcome assessors were ward nurses recording pain scores and analgesic use. The statistical analyst was not blinded but received anonymized datasets.

**Reviewer #2**

**1. Trial registration**

**Response**: Retrospective registration is acknowledged and justified (administrative delays and a shortage of stable internet access). Ethical approval was obtained prior to recruitment.

**2. Randomization and baseline imbalance**

**Response**: We agree that these imbalances are unexpected in randomized trials. Randomization procedure clarified; regression analyses performed to adjust for imbalances.

**3. Sample size calculation and power**

**Response**: We thank the reviewer for highlighting this. Rationale for enrolling 30 per group added (precision, external validity, attrition). Primary outcome powered; secondary outcomes exploratory. This is explicitly stated in the Method section.

**4. Blinding**

**Response**: We acknowledge partial blinding as a limitation. IV dexamethasone and saline were prepared identically by an independent nurse. This is clarified in the Method section.

**5. Statistical analysis and multiple comparisons**

**Response**: We appreciate this observation. Pain scores re‑analyzed with repeated‑measures ANOVA. 24‑hour scores not compared due to widespread rescue analgesia.

**6. Primary outcome definition**

**Response**: Duration of analgesia defined as time from block completion to first rescue analgesia or NRS ≥4. Thresholds standardized.

**Minor: CONSORT reporting**

**Response**: Sequence generation and envelope handling clarified. Flow diagram referenced consistently.

**Minor: Adverse events**

**Response**: We have added a structured table summarizing monitored adverse events in the result Section of the revised manuscript.

**Minor: Language**

**Response**: We have thoroughly revised the manuscript for grammar, clarity and readability. Short, fragmented paragraph have been merged, and repetition reduced. The revised version has undergone language editing.

**Reviewer #3**

**1. Hypothesis**

**Response**: We agree. We have now explicitly stated our hypothesis at the end of the Introduction.

**2. Randomization details**

**Response**: We have expanded the Methods section to describe the randomization process in detail. Sequence generation, envelope preparation, and allocation concealment described in detail.

**3. Retrospective registration**

**Response**: We acknowledge this limitation. Justification is now provided in the Method section and the limitation is acknowledged.

**4. Sample size calculation**

**Response**: Units clarified (hours), reference study cited, rationale for 30 per group explained.

**5. Outcome definitions and consistency**

**Response**: Duration of analgesia defined; NRS terminology standardized; rationale for omission of 24‑hour analysis explained.

**6. Statistical analysis clarity**

**Response**: We have revised the Statistical Analysis section to explicitly state which variables were compared using independent t-tests, Mann–Whitney U tests, chi-square tests, or regression models. Each parameter and test explicitly stated.

**7. Language and grammar**

Response: The manuscript has undergone thorough language editing. Errors such as “the effective of dexamethasone” have been corrected. We have improved clarity, reduced redundancy, and ensured professional tone throughout. Manuscript professionally edited.

**8. Abbreviations**

**Response**: All abbreviations (e.g., SCBPB, NRS, ASA) are now defined at first mention and used consistently thereafter. All abbreviations defined at first mention and used consistently.

**9. Figures and tables**

**Response**: We have revised all figures to include clear legends, units, and consistent numbering. Figures renumbered, legends clarified, redundant data removed, tables reorganized.

**10. Discussion**

**Response**: We have substantially revised the Discussion. The Discussion section is revised to interpret results, include mean ± SD, compare with literature, emphasize novelty of intravenous dexamethasone, and acknowledge limitations.

**Editorial Office Requirements**

**Style**: Manuscript reformatted per PLOS ONE templates.

**Trial registration**: Registry name, number, and recruitment/follow‑up dates included on title page and in Methods. Retrospective registration justified. Statement added: “The authors confirm that all ongoing and related trials for this intervention are registered.”

**Data availability**: Updated statement. Restriction explained with ethics committee contact.

**Ethics statement**: Appears only in Methods.

**Figures/tables**: CONSORT flow diagram uploaded; figure numbering corrected; all tables cited in text.

**Supporting Information**: Captions added and citations updated.

**Conclusion**

We believe these revisions address all reviewer and editorial concerns. The manuscript has been strengthened in methodology, transparency, and clarity, and we hope it is now suitable for publication in PLOS ONE.
