# Supplementary material for: The effect of intravenous dexamethasone in supraclavicular brachial plexus block at Halibet National Referral Hospital, Eritrea 2024–2025: A randomized clinical trial
Source: PLoS One. 2026 Sep 3;21(9):e0338067. doi: 10.1371/journal.pone.0338067 (PMC13541117; doi:10.1371/journal.pone.0338067)
Supplement: Supplementary file 13 [file pone.0338067.s007.docx]

**June 17, 2026**

**Response to Reviewers**

We thank the Academic Editor and Reviewers for their continued constructive feedback. We have carefully revised the manuscript to address each point raised. Below, we provide point‑by‑point responses.

**Reviewer #1**

- Statistical assumptions

Response: We now explicitly report that Shapiro‑Wilk and Levene’s tests were performed to confirm normality and homogeneity of variance prior to parametric analyses. These results are added to the Methods section.

- Regression reporting

Response: We have expanded the Results to include regression coefficients, adjusted effect estimates, and confidence intervals. A supplementary table has been added to allow readers to assess the impact of baseline imbalances.

**Reviewer #5**

- Acknowledgment of strengths

Response: We thank the reviewer for recognizing the clinical relevance of dexamethasone in pain management. The analgesics table has been proofread and clarified for consistency.

**Reviewer #6**

- Sample size calculation

Response: We acknowledge the concern regarding circularity in variance estimates. We have added a sensitivity analysis and post‑hoc power commentary for secondary outcomes. This strengthens transparency around the adequacy of our sample size.

- Trial registration

Response: Retrospective registration remains a limitation. We have expanded the explanation in the Methods and Discussion, and explicitly acknowledge the risk of reporting bias.

- Blinding

Response: We clarify that ward nurses recording NRS scores were not aware of group allocation, but they were aware that patients were enrolled in a study. We have added a statement in the Discussion acknowledging that this partial blinding may introduce expectation bias and recommending full blinding of outcome assessors in future trials.

- Time‑to‑event analysis for the 24‑hour NRS data

Response: We acknowledge this as a valuable approach. However, due to widespread rescue analgesia use within 24 hours, the data were heavily censored, limiting feasibility. We therefore analyzed pain scores at standardized intervals using repeated‑measures ANOVA and t‑tests, which allowed consistent comparison.

- Incomplete references

Response: All references (4, 7, 9, 10, 13, 14, 17, 20, 21, 22, 23, and 24) have been corrected with complete bibliographic details. Reference 12 has been corrected with a primary pharmacology source for dexamethasone’s mechanism.

- Typographic errors

Response: Recruitment dates corrected to “September 23, 2024 to January 17, 2025.” The contribution statement typo (“ollection”) has been fixed.

- Generalizability

Response: We now explicitly acknowledge that all enrolled patients were ASA I, which may limit generalizability to higher‑risk populations. We have included in the discussion section of the manuscript.

**Closing**

We believe these revisions address all outstanding concerns. The manuscript now includes strengthened statistical reporting, clarified blinding procedures, corrected references, and explicit acknowledgment of limitations. We hope this revised version is suitable for publication in PLOS ONE.
