# Supplementary material for: The effect of intravenous dexamethasone in supraclavicular brachial plexus block at Halibet National Referral Hospital, Eritrea 2024–2025: A randomized clinical trial
Source: PLoS One. 2026 Sep 3;21(9):e0338067. doi: 10.1371/journal.pone.0338067 (PMC13541117; doi:10.1371/journal.pone.0338067)
Supplement: Supplementary file 14 [file pone.0338067.s008.docx]

**July 22, 2026**

**Response to Reviewers**

We thank the Academic Editor and Reviewers for their continued constructive feedback. We have carefully revised the manuscript to address each point raised. Below, we provide point‑by‑point responses. We respectfully note that many of the issues raised in this round overlap with concerns already addressed in our previous revision, and we highlight where these clarifications are present in the manuscript.

**Reviewer #7**

- **Abstract: inclusion criteria and outcomes**

Response: We explicitly write the polished and birds-eye view note of the manuscript in the abstract section (abstract section, page 2). The inclusion criteria (ASA I patients, elective orthopedic upper limb surgery, age range 18–80) are clearly stated in the Methods. Primary outcome (duration of analgesia) and secondary outcomes (onset of block, pain scores, analgesic consumption, adverse events) is presented in the Methods section.

- **References or citations in introduction section**

Response: We appreciate for the candid observation, but we have included some newer published articles (Nwosu et al. 2023, Edinoff et al. 2021, Hewson et al. 2019). These are cited in the introduction. Some of the relatively old articles have strong scientific values and we don’t want to ignore it.

- **Materials and Methods**

**Response**: We respectfully note that the reviewer to read the method section, as outlined below.

**Drug details**: 0.5% bupivacaine (20 mL) and intravenous dexamethasone (8 mg) specified and most of drugs in low setting environments such as Eritrea, are manufactured from India. (Method sec, page: 16–17).

**Side effects**: Hypotension, bradycardia, respiratory depression, nausea, vomiting, allergic reactions, seizures monitored and reported in Table 4 of the result section (page, 22–23).

**Group matching**: Type of surgery (skeletal vs soft tissue, elective only) clearly clarified in Methods (page, 14-15).

**Procedure technique**: A detailed description of the techniques used can be found in the Methods. Ultrasound nerve block is always performed in supine position, and this is a supraclavicular brachial plexus block where the landmark is clear. In-plane approach was performed (page, 15–16).

**Randomization and blinding**: Sealed envelope randomization and partial blinding explained (page, 16).

**Postoperative pain management**: NRS‑based analgesia protocol with diclofenac, ibuprofen, and paracetamol described (page, 16 and page, 20-21).

- **Results – side effects, analgesic usage, figures**

**Response:**

**Side effects**: Table 4 (page, 22–23) shows no adverse events in either group.

**Analgesic usage**: Table 3 (page, 21) details diclofenac, ibuprofen, and paracetamol consumption.

**Figures**: Hemodynamic parameters presented in Figure 2 (page, 20); pain scores in Figure 3 (page, 21).

**Discussion** – pharmacology of dexamethasone

**Response**: Pharmacokinetic and pharmacodynamic mechanisms of dexamethasone (anti‑inflammatory properties, modulation of nociceptive transmission, onset/duration effects) discussed with references (page, 23-24).

- **References**

**Response**: Updated references (2019–2023) added (page, 26-28).

**Reviewer #8**

- **Title clarity**

**Response**: We have retained the phrasing “The effects of intravenous dexamethasone in supraclavicular brachial plexus block” because the study specifically measured and reported outcomes – duration of analgesia, onset of block, analgesia consumption, and adverse events. The phrase “effects of” emphasizes that this was an outcome-focused randomized clinical trial, rather than a descriptive report of the intervention itself. This distinction aligns the title with the study’s design and objectives, highlighting that the manuscript contributes evidence on measurable clinical effects rather than simply describing the use of dexamethasone.

**Abstract clarity**

**Response**: Thank you for the comment but the abstract section is written to provide a highlight of the study. The detailed component of the study is expressed clearly in the whole part of the manuscript.

- **Methods** – timing and drug administration

**Response**: Methods specify SCBPB performed with 20 mL of 0.5% bupivacaine; control group received 2 mL normal saline IV, experimental group received 8 mg dexamethasone IV

**Closing**

We believe these revisions address all outstanding concerns from Reviewer #7 and Reviewer #8. The manuscript has been revised and polished to meet the criteria of PLOS ONE. Most of the comments provided by the recent reviewers were already addressed in the previous cycle of revision, and we have highlighted where these clarifications are present. Given the extensive revisions and multiple independent reviews already completed, we respectfully request that the editor consider this version suitable for publication in PLOS ONE.
