# Supplementary material for: The effect of intravenous dexamethasone in supraclavicular brachial plexus block at Halibet National Referral Hospital, Eritrea 2024–2025: A randomized clinical trial
Source: PLoS One. 2026 Sep 3;21(9):e0338067. doi: 10.1371/journal.pone.0338067 (PMC13541117; doi:10.1371/journal.pone.0338067)
Supplement: Peer review history

# Peer review: Decision Letter 0

**DOI:** 10.1371/journal.pone.0338067.r001

**Authors:** Robert Jeenchen Chen

22 Jan 2026

Dear Dr. Mengistu,

Please submit your revised manuscript by Mar 08 2026 11:59PM. If you will need more time than this to complete your revisions, please reply to this message or contact the journal office at plosone@plos.org. When you're ready to submit your revision, log on to https://www.editorialmanager.com/pone/ and select the 'Submissions Needing Revision' folder to locate your manuscript file.

Please include the following items when submitting your revised manuscript:

- A letter that responds to each point raised by the academic editor and reviewer(s). You should upload this letter as a separate file labeled 'Response to Reviewers'.
- A marked-up copy of your manuscript that highlights changes made to the original version. You should upload this as a separate file labeled 'Revised Manuscript with Track Changes'.
- An unmarked version of your revised paper without tracked changes. You should upload this as a separate file labeled 'Manuscript'.

If you would like to make changes to your financial disclosure, please include your updated statement in your cover letter. Guidelines for resubmitting your figure files are available below the reviewer comments at the end of this letter.

If applicable, we recommend that you deposit your laboratory protocols in protocols.io to enhance the reproducibility of your results. Protocols.io assigns your protocol its own identifier (DOI) so that it can be cited independently in the future. For instructions see: https://journals.plos.org/plosone/s/submission-guidelines#loc-laboratory-protocols. Additionally, PLOS ONE offers an option for publishing peer-reviewed Lab Protocol articles, which describe protocols hosted on protocols.io. Read more information on sharing protocols at https://plos.org/protocols?utm_medium=editorial-email&utm_source=authorletters&utm_campaign=protocols.

We look forward to receiving your revised manuscript.

Kind regards,

Robert Jeenchen Chen, MD, MPH, ChFC®, EA

Academic Editor

PLOS One

Journal Requirements:

When submitting your revision, we need you to address these additional requirements.

1. Please ensure that your manuscript meets PLOS ONE's style requirements, including those for file naming. The PLOS ONE style templates can be found at

https://journals.plos.org/plosone/s/file?id=wjVg/PLOSOne_formatting_sample_main_body.pdf and

https://journals.plos.org/plosone/s/file?id=ba62/PLOSOne_formatting_sample_title_authors_affiliations.pdf

2. We note that you have selected “Clinical Trial” as your article type. PLOS ONE requires that all clinical trials are registered in an appropriate registry (the WHO list of approved registries is at https://www.who.int/clinical-trials-registry-platform/network/primary-registries" https://www.who.int/clinical-trials-registry-platform/network/primary-registries and more information on trial registration is at http://www.icmje.org/about-icmje/faqs/clinical-trials-registration/).

Please state the name of the registry and the registration number (e.g. ISRCTN or ClinicalTrials.gov) in the submission data and on the title page of your manuscript.

a) Please provide the complete date range for participant recruitment and follow-up in the methods section of your manuscript.

b) If you have not yet registered your trial in an appropriate registry, we now require you to do so and will need confirmation of the trial registry number before we can pass your paper to the next stage of review. Please include in the Methods section of your paper your reasons for not registering this study before enrolment of participants started. Please confirm that all related trials are registered by stating: “The authors confirm that all ongoing and related trials for this drug/intervention are registered.”

Please see http://journals.plos.org/plosone/s/submission-guidelines#loc-clinical-trials for our policies on clinical trials.

3. We note that you have indicated that there are restrictions to data sharing for this study. For studies involving human research participant data or other sensitive data, we encourage authors to share de-identified or anonymized data. However, when data cannot be publicly shared for ethical reasons, we allow authors to make their data sets available upon request. For information on unacceptable data access restrictions, please see http://journals.plos.org/plosone/s/data-availability#loc-unacceptable-data-access-restrictions.

Before we proceed with your manuscript, please address the following prompts:

a) If there are ethical or legal restrictions on sharing a de-identified data set, please explain them in detail (e.g., data contain potentially identifying or sensitive patient information, data are owned by a third-party organization, etc.) and who has imposed them (e.g., a Research Ethics Committee or Institutional Review Board, etc.). Please also provide contact information for a data access committee, ethics committee, or other institutional body to which data requests may be sent.

b) If there are no restrictions, please upload the minimal anonymized data set necessary to replicate your study findings to a stable, public repository and provide us with the relevant URLs, DOIs, or accession numbers. Please see http://www.bmj.com/content/340/bmj.c181.long for guidelines on how to de-identify and prepare clinical data for publication. For a list of recommended repositories, please see https://journals.plos.org/plosone/s/recommended-repositories. You also have the option of uploading the data as Supporting Information files, but we would recommend depositing data directly to a data repository if possible.

Please update your Data Availability statement in the submission form accordingly.

4. In the online submission form, you indicated that [The datasets generated and/or analyzed during the current study are available from the corresponding author on reasonable request].

All PLOS journals now require all data underlying the findings described in their manuscript to be freely available to other researchers, either 1. In a public repository, 2. Within the manuscript itself, or 3. Uploaded as supplementary information.

This policy applies to all data except where public deposition would breach compliance with the protocol approved by your research ethics board. If your data cannot be made publicly available for ethical or legal reasons (e.g., public availability would compromise patient privacy), please explain your reasons on resubmission and your exemption request will be escalated for approval.

5. Your ethics statement should only appear in the Methods section of your manuscript. If your ethics statement is written in any section besides the Methods, please delete it from any other section.

6. Please upload a copy of Figure 1, to which you refer in your text on pages 7 and 11. If the figure is no longer to be included as part of the submission please remove all reference to it within the text.

7. We note that you have included two Figure 1’s in your manuscript. Please clarify the captions of these figures accordingly to match the citations that were provided in your manuscript.

8. Figure 2 is not cited in the text. Please review and ensure the Figure is properly cited where necessary.

9. We note you have included a table to which you do not refer in the text of your manuscript. Please ensure that you refer to Tables 3, 4, 5, and 6 in your text; if accepted, production will need this reference to link the reader to the tables.

10. Please include captions for your Supporting Information files at the end of your manuscript, and update any in-text citations to match accordingly. Please see our Supporting Information guidelines for more information: http://journals.plos.org/plosone/s/supporting-information.

If the reviewer comments include a recommendation to cite specific previously published works, please review and evaluate these publications to determine whether they are relevant and should be cited. There is no requirement to cite these works unless the editor has indicated otherwise.

[Note: HTML markup is below. Please do not edit.]

Reviewers' comments:

Reviewer's Responses to Questions

**Comments to the Author**

1. Is the manuscript technically sound, and do the data support the conclusions?

Reviewer #1: Partly

Reviewer #2: Partly

Reviewer #3: Partly

**********

2. Has the statistical analysis been performed appropriately and rigorously?

Reviewer #1: Yes

Reviewer #2: Yes

Reviewer #3: Yes

**********

3. Have the authors made all data underlying the findings in their manuscript fully available?

The PLOS Data policy

Reviewer #1: Yes

Reviewer #2: Yes

Reviewer #3: Yes

**********

4. Is the manuscript presented in an intelligible fashion and written in standard English?

Reviewer #1: Yes

Reviewer #2: No

Reviewer #3: No

**********

Reviewer #1: As the statistical reviewer I will focus on methods and reporting.

Major

1) The power calculations need to be presented after the primary outcome has been defined. Then about the power calculations: what was the previous study on which the power calculation was based? it is not referenced. It is unusual for just high variation in the two groups, was that other study large enough? what is mu1 and mu2 in this case? minutes? days? hours? The assumption is for a massive effect, which is problematic when it comes to an RCT

2) the authors are effectively conducting a non-inferiority trial (not a comparison with placebo) without having approached the design as such. This is a major limitation that needs to be clearly discussed since nothing else can be done about it at this late stage.

3) I was not impressed by the structure and quality of writing - reminded me a lot of AI output with bullet points, repetition and very short paragraphs, although that I cannot prove.

4) how did the authors deal with missing data? this needs to be clearly stated in the paper - were all data complete? if yest please state so.

5) there is great variation in characteristics, sand I wouldn't expect that to be the case when randomised properly - the chances of such imbalances are pretty small. that is pretty worrying and also means that regression models need to be used to account for the imbalances, which may be problematic with such a small sample size.

6) linked to 5, move to a regression analysis framework so effect sizes and confidence intervals can be reported, not just p-values. normally, i'd expect a simple regresison with no coavariates except the intervention, but here there exist large imbalances.

7) The trial was registered after completion, which is a major transparency issue

Minor

1) "Outcome assessors were blinded". who were these? the statistical analyst?

Reviewer #2: General Comment

This randomized clinical trial evaluates the effect of intravenous dexamethasone as an adjunct to bupivacaine in supraclavicular brachial plexus block for upper-limb surgery. The topic is clinically relevant, particularly in low-resource settings, and the study reports large and consistent effects on analgesia duration and block characteristics. However, several methodological, reporting, and compliance issues must be addressed to ensure transparency, reproducibility, and adherence to PLOS ONE standards.

Major Comments

1. Trial Registration

The trial was registered retrospectively in the Pan African Clinical Trial Registry (PACTR202509579633417), after participant recruitment and data collection were completed.

• Explicitly justify the reason for retrospective registration.

2. Randomization and Baseline Imbalance

Despite randomization, statistically significant baseline differences were observed between groups in:

• Religion (p < 0.001)

• Ethnicity (p = 0.004)

• BMI (p = 0.039)

These imbalances raise concerns regarding the effectiveness of the randomization process and may confound outcomes, particularly BMI, which can affect pharmacokinetics and pain perception.

3. Sample Size Calculation and Power

The sample size calculation indicates a minimum of 7 participants per group, yet 30 participants per group were enrolled without a clear justification.

• Provide a clear rationale for enrolling 30 participants per group (e.g., anticipated attrition, external validity, precision of estimates).

• Specify whether the study was powered for secondary outcomes (onset times, pain scores, analgesic consumption).

4. Blinding and Risk of Bias

The anesthetist performing the block was not blinded, while participants and outcome assessors were blinded.

• Discuss the potential impact of partial blinding in the Limitations section.

• Clarify whether IV dexamethasone and normal saline were prepared and administered in an identical manner by an independent staff member.

5. Statistical Analysis and Multiple Comparisons

Pain scores were compared at multiple postoperative time points without adjustment for multiple testing. In addition, 24-hour pain scores were reported descriptively but not statistically analyzed.

Repeated unadjusted comparisons increase the risk of Type I error.

• Justify the absence of correction for multiple comparisons or apply an appropriate method (e.g., repeated-measures analysis).

• Clarify why 24-hour pain scores were not statistically compared (e.g., widespread rescue analgesia).

6. Definition of Primary Outcome

“Duration of postoperative analgesia” is not operationally defined.

• Explicitly define duration of analgesia (e.g., time from block completion to first request for rescue analgesia or NRS ≥4).

• State whether analgesic administration thresholds were standardized.

Minor Comments

7. CONSORT Reporting

Although CONSORT adherence is stated, key methodological details (sequence generation, allocation concealment) require clearer description.

• Expand the randomization subsection to explicitly describe sequence generation and envelope handling.

• Ensure the CONSORT flow diagram is clearly referenced and consistent with the text.

8. Adverse Events Reporting

No adverse events were reported, but this is presented narratively without structured data.

• Add a table summarizing monitored adverse events

9. Language and Editorial Issues

Several grammatical and stylistic errors are present throughout the manuscript.

• Perform thorough language editing to improve clarity and readability.

Overall Recommendation

Major Revision

The manuscript addresses an important clinical issue and shows promising results, but methodological clarification, improved statistical reporting, and compliance with PLOS ONE policies are required before it can be considered for publication.

Reviewer #3: ________________________________________

In this study, authors investigated the effect of intravenous dexamethasone combined with bupivacaine on prolonging postoperative analgesia following supraclavicular brachial plexus block. They reported that the addition of 8 mg intravenous dexamethasone to 0.5% bupivacaine in supraclavicular brachial plexus blocks significantly prolonged postoperative analgesia, accelerated the onset of sensory and motor block, and reduced analgesic consumption.

The following major revisions are necessary for this study:

-What is the authors' hypothesis? On what basis did they conclude that intravenous dexamethasone might improve the success of the block? The hypothesis should be written at the end of the introduction, and the argument should be written based on that hypothesis.

- The randomization process should be described in detail, specifying at what stage the patients were randomized. Clarify how the random sequence was generated.Specify who prepared the sealed opaque envelopes and how allocation concealment was ensured. Explicitly state whether block randomization or stratification was considered.

-The trial was registered retrospectively, which is a major limitation for a randomized clinical trial. The authors should: provide a clear justification for retrospective registration. Explicitly acknowledge retrospective registration as a limitation.

-There's a problem with calculating the sample size. Getting 7 is unlikely. It needs to be recalculated. The sample size calculation suggests that only 7 participants per group were required, yet 30 were included in each group. While over-recruitment can be acceptable, the rationale for this decision is not adequately justified. Additionally: The reported means (μ₁ and μ₂) appear unusually large and their units are unclear. The reference study used for variance estimation should be clearly cited and explained.

-Outcome Definitions and Measurement Consistency: The definition of “duration of analgesia” should be clarified (e.g., time from block completion to first request for rescue analgesia). Pain at 24 hours is reported descriptively but not statistically compared; the rationale for this omission should be explained. The use of NRS is appropriate, but the manuscript alternates between NRS and VAS terminology, which should be standardized.

-Standard statistical analysis statements should not be written; it should be clearly stated which parameter was compared with which test.

-Many grammatical errors and awkward phrasings are present (e.g., “the effective of dexamethasone” instead of “the effect of dexamethasone”). Professional language editing is strongly recommended.

-Clarify abbreviations at first mention and avoid redundancy.

-Figures should include clear legends and units. Figure numbering appears inconsistent (e.g., Figure 1 vs. Figure 2 in the text). Consort flow diagram??

-It is unnecessary to present the same data in both figures and tables.

- Parameters like ‘Educational level, Religious affiliation, Ethnicity’ are unnecessary as they do not affect the study results. The total column in Tables 1 and 2 is also unnecessary. Data can be presented in a single row; simply stating male/female or the number of individuals with an anesthesia history would suffice. The tables should be reorganized, and Tables 1 and 2 should be merged.

-Table 3's RR and spo2 are unnecessary. Changes in blood pressure should be presented graphwise; a table is not needed. Table 6 should be rearranged, and Table 5 and Figure 3 should be shown only as figures, with data written below the figure. Figure 1?

-The discussion section should include figures and mean-standard deviations. The discussion should be a section interpreting the results. The discussion should be revised to include the results of studies in the literature and the factors influencing the differences in results. The new contribution of this study to the literature should be stated.

-The discussion occasionally overstates causality given the study limitations. Comparisons with existing literature are appropriate but could be more concise. The novelty of intravenous (rather than perineural) dexamethasone in this specific setting should be more clearly emphasized.

In conclusion, major revisions are necessary.

Sincerely yours…

**********

what does this mean?). If published, this will include your full peer review and any attached files.

If you choose “no”, your identity will remain anonymous but your review may still be made public.

**Do you want your identity to be public for this peer review?**  For information about this choice, including consent withdrawal, please see our Privacy Policy

Reviewer #1: No

Reviewer #2: No

Reviewer #3: No

**********

[NOTE: If reviewer comments were submitted as an attachment file, they will be attached to this email and accessible via the submission site. Please log into your account, locate the manuscript record, and check for the action link "View Attachments". If this link does not appear, there are no attachment files.]

To ensure your figures meet our technical requirements, please review our figure guidelines: https://journals.plos.org/plosone/s/figures

You may also use PLOS’s free figure tool, NAAS, to help you prepare publication quality figures: https://journals.plos.org/plosone/s/figures#loc-tools-for-figure-preparation.

NAAS will assess whether your figures meet our technical requirements by comparing each figure against our figure specifications.

Attachment Submitted filename: Reviewer comments.docx

# Author comment: Author response to Decision Letter 1

**DOI:** 10.1371/journal.pone.0338067.r002

2 Feb 2026

Please see the attached detailed response‑to‑reviewers letter. We have carefully addressed each reviewer and editor comment point‑by‑point, with corresponding revisions incorporated into the tracked‑changes and clean versions of the manuscript. Figures and tables have been updated, captions standardized, and all methodological clarifications added as requested. We believe the revised submission fully addresses the concerns raised and strengthens the manuscript.

Attachment Submitted filename: Response to reviewers.docx

# Peer review: Decision Letter 1

**DOI:** 10.1371/journal.pone.0338067.r003

**Authors:** Robert Jeenchen Chen

25 Mar 2026

Dear Dr. Mengistu,

Thank you for submitting your manuscript to PLOS ONE. After careful consideration, we feel that it has merit but does not fully meet PLOS ONE’s publication criteria as it currently stands. Therefore, we invite you to submit a revised version of the manuscript that addresses the points raised during the review process.

Please submit your revised manuscript by May 09 2026 11:59PM. If you will need more time than this to complete your revisions, please reply to this message or contact the journal office at plosone@plos.org. When you're ready to submit your revision, log on to https://www.editorialmanager.com/pone/ and select the 'Submissions Needing Revision' folder to locate your manuscript file.

- A letter that responds to each point raised by the academic editor and reviewer(s). You should upload this letter as a separate file labeled 'Response to Reviewers'.
- A marked-up copy of your manuscript that highlights changes made to the original version. You should upload this as a separate file labeled 'Revised Manuscript with Track Changes'.
- An unmarked version of your revised paper without tracked changes. You should upload this as a separate file labeled 'Manuscript'.

If you would like to make changes to your financial disclosure, please include your updated statement in your cover letter. Guidelines for resubmitting your figure files are available below the reviewer comments at the end of this letter.

If applicable, we recommend that you deposit your laboratory protocols in protocols.io to enhance the reproducibility of your results. Protocols.io assigns your protocol its own identifier (DOI) so that it can be cited independently in the future. For instructions see: https://journals.plos.org/plosone/s/submission-guidelines#loc-laboratory-protocols. Additionally, PLOS ONE offers an option for publishing peer-reviewed Lab Protocol articles, which describe protocols hosted on protocols.io. Read more information on sharing protocols at https://plos.org/protocols?utm_medium=editorial-email&utm_source=authorletters&utm_campaign=protocols.

As the corresponding author, your ORCID iD is verified in the submission system and will appear in the published article. PLOS supports the use of ORCID, and we encourage all coauthors to register for an ORCID iD and use it as well. Please encourage your coauthors to verify their ORCID iD within the submission system before final acceptance, as unverified ORCID iDs will not appear in the published article. *Only*  the individual author can complete the verification step; PLOS staff *cannot*  verify ORCID iDs on behalf of authors.

We look forward to receiving your revised manuscript.

Kind regards,

Robert Jeenchen Chen, MD, MPH, ChFC®, EA

Academic Editor

PLOS One

Journal Requirements:

If the reviewer comments include a recommendation to cite specific previously published works, please review and evaluate these publications to determine whether they are relevant and should be cited. There is no requirement to cite these works unless the editor has indicated otherwise.

[Note: HTML markup is below. Please do not edit.]

Reviewers' comments:

Reviewer's Responses to Questions

**Comments to the Author**

Reviewer #1: (No Response)

Reviewer #4: All comments have been addressed

**********

2. Is the manuscript technically sound, and do the data support the conclusions?

Reviewer #1: Partly

Reviewer #4: Yes

**********

3. Has the statistical analysis been performed appropriately and rigorously?

Reviewer #1: No

Reviewer #4: I Don't Know

**********

4. Have the authors made all data underlying the findings in their manuscript fully available?

The PLOS Data policy

Reviewer #1: Yes

Reviewer #4: Yes

**********

5. Is the manuscript presented in an intelligible fashion and written in standard English?

Reviewer #1: Yes

Reviewer #4: Yes

**********

Reviewer #1: I am still not comfortable with this work and I don't need to see it again. I expected my original comments unaltered and the response underneath followed by the resulting changes to the paper, but that was not the case. my original concerns remain in the large.

Reviewer #4: - in the intervention section, I would make sure that the intervention in the abstract & methods section agree (ie. potentially including that IV saline was given to the control group)

- discussion: instead of "confirming our hypothesis that systemic corticosteroid administration enhances block quality and duration," I suggest a verb more similar to "supporting"

- potentially creating a separate subsection for strengths/limitations

**********

what does this mean?). If published, this will include your full peer review and any attached files.

If you choose “no”, your identity will remain anonymous but your review may still be made public.

**Do you want your identity to be public for this peer review?**  For information about this choice, including consent withdrawal, please see our Privacy Policy

Reviewer #1: No

Reviewer #4: No

**********

[NOTE: If reviewer comments were submitted as an attachment file, they will be attached to this email and accessible via the submission site. Please log into your account, locate the manuscript record, and check for the action link "View Attachments". If this link does not appear, there are no attachment files.]

To ensure your figures meet our technical requirements, please review our figure guidelines: https://journals.plos.org/plosone/s/figures

You may also use PLOS’s free figure tool, NAAS, to help you prepare publication quality figures: https://journals.plos.org/plosone/s/figures#loc-tools-for-figure-preparation.

NAAS will assess whether your figures meet our technical requirements by comparing each figure against our figure specifications.

# Author comment: Author response to Decision Letter 2

**DOI:** 10.1371/journal.pone.0338067.r004

1 Apr 2026

We recognize Reviewer #1’s continued reservations. While the completed trial design cannot be altered, we have strengthened our statistical analysis by clarifying the primary outcome definition, adjusting for baseline imbalances through regression analyses, and framing conclusions more cautiously. We hope these revisions demonstrate our diligence and transparency.

Reviewer #4:

We have revised the Abstract and Methods to explicitly state that the control group received intravenous saline, ensuring consistency across sections. In the Discussion, we replaced “confirming our hypothesis” with “supporting our hypothesis” to reflect a more cautious interpretation. Finally, we created a dedicated subsection titled “Strengths and Limitations,” highlighting methodological rigor and acknowledging study constraints such as retrospective registration, partial blinding, and baseline imbalances.

Attachment Submitted filename: Response_to_reviewers_auresp_2.docx

# Peer review: Decision Letter 2

**DOI:** 10.1371/journal.pone.0338067.r005

**Authors:** Robert Jeenchen Chen

10 Jun 2026

Dear Dr. Mengistu,

Thank you for submitting your manuscript to PLOS ONE. After careful consideration, we feel that it has merit but does not fully meet PLOS ONE’s publication criteria as it currently stands. Therefore, we invite you to submit a revised version of the manuscript that addresses the points raised during the review process.

Revise.

Please submit your revised manuscript by Jul 25 2026 11:59PM. If you will need more time than this to complete your revisions, please reply to this message or contact the journal office at plosone@plos.org. When you're ready to submit your revision, log on to https://www.editorialmanager.com/pone/ and select the 'Submissions Needing Revision' folder to locate your manuscript file.

- A letter that responds to each point raised by the academic editor and reviewer(s). You should upload this letter as a separate file labeled 'Response to Reviewers'.
- A marked-up copy of your manuscript that highlights changes made to the original version. You should upload this as a separate file labeled 'Revised Manuscript with Track Changes'.
- An unmarked version of your revised paper without tracked changes. You should upload this as a separate file labeled 'Manuscript'.

If you would like to make changes to your financial disclosure, please include your updated statement in your cover letter. Guidelines for resubmitting your figure files are available below the reviewer comments at the end of this letter.

If applicable, we recommend that you deposit your laboratory protocols in protocols.io to enhance the reproducibility of your results. Protocols.io assigns your protocol its own identifier (DOI) so that it can be cited independently in the future. For instructions see: https://journals.plos.org/plosone/s/submission-guidelines#loc-laboratory-protocols. Additionally, PLOS ONE offers an option for publishing peer-reviewed Lab Protocol articles, which describe protocols hosted on protocols.io. Read more information on sharing protocols at https://plos.org/protocols?utm_medium=editorial-email&utm_source=authorletters&utm_campaign=protocols.

As the corresponding author, your ORCID iD is verified in the submission system and will appear in the published article. PLOS supports the use of ORCID, and we encourage all coauthors to register for an ORCID iD and use it as well. Please encourage your coauthors to verify their ORCID iD within the submission system before final acceptance, as unverified ORCID iDs will not appear in the published article. *Only*  the individual author can complete the verification step; PLOS staff *cannot*  verify ORCID iDs on behalf of authors.

We look forward to receiving your revised manuscript.

Kind regards,

Robert Jeenchen Chen, MD, MPH, ChFC®, EA

Academic Editor

PLOS One

Journal Requirements:

If the reviewer comments include a recommendation to cite specific previously published works, please review and evaluate these publications to determine whether they are relevant and should be cited. There is no requirement to cite these works unless the editor has indicated otherwise.

[Note: HTML markup is below. Please do not edit.]

Reviewers' comments:

Reviewer's Responses to Questions

**Comments to the Author**

Reviewer #1: (No Response)

Reviewer #5: (No Response)

Reviewer #6: All comments have been addressed

**********

2. Is the manuscript technically sound, and do the data support the conclusions?

Reviewer #1: Partly

Reviewer #5: Yes

Reviewer #6: Partly

**********

3. Has the statistical analysis been performed appropriately and rigorously?

Reviewer #1: No

Reviewer #5: Yes

Reviewer #6: No

**********

4. Have the authors made all data underlying the findings in their manuscript fully available?

The PLOS Data policy

Reviewer #1: Yes

Reviewer #5: (No Response)

Reviewer #6: No

**********

5. Is the manuscript presented in an intelligible fashion and written in standard English?

Reviewer #1: (No Response)

Reviewer #5: Yes

Reviewer #6: Yes

**********

Reviewer #1: nothing further to add...............................................................................

Reviewer #5: Thank you for your work on the interesting paper about the impact of dexamethasone on pain management. Dexamethasone, being a corticosteroid does reduce inflammation, which is correlated with pain. This is indicated from the analgesics table. Your results supports that further.

Reviewer #6: The authors present a randomized clinical trial evaluating the effect of a single 8 mg intravenous dose of dexamethasone as an adjunct to bupivacaine in supraclavicular brachial plexus block (SCBPB) for upper limb surgery. The topic is clinically relevant, particularly in resource-constrained settings where alternative analgesic strategies—such as continuous perineural catheters or neuraxial opioids—carry practical limitations. The investigators are to be commended for undertaking this work in a setting where such data have historically been sparse. That said, there remain methodological issues that I believe warrant consideration before the manuscript advances.

Strengths

The most significant strength of this work is its clinical context. Conducting a prospective randomized trial at a single tertiary referral hospital in Eritrea fills a genuine gap in the regional literature, and the authors are transparent about this contribution. The primary outcome—duration of postoperative analgesia, defined clearly as time from block completion to first rescue analgesia request or NRS ≥ 4—is appropriate and operationalized consistently across both arms. The analgesia duration results (17.36 ± 3.75 hours in the dexamethasone group versus 7.57 ± 1.04 hours in controls, p < 0.001) are large in magnitude and closely replicate estimates from prior work cited by the authors (Pawan et al., 2019; Saba et al., 2020), lending face validity to the findings.

The revised manuscript also benefits from the addition of a dedicated Strengths and Limitations subsection, improved consistency in reporting the control group's IV saline administration, and the more measured language in the Discussion ("supporting" rather than "confirming" the hypothesis). These changes reflect a genuine responsiveness to reviewer feedback. The CONSORT flow diagram and the structured adverse events table are appropriate additions.

Statistical Appropriateness

The choice of independent-samples t-tests for the primary outcome is reasonable given the sample sizes and the apparent normality assumptions, though the manuscript would benefit from explicitly reporting whether Shapiro-Wilk or Levene's tests were performed prior to parametric analyses, particularly given the asymmetric SDs (e.g., the experimental group's analgesia duration SD of 3.75 hours vs. 1.04 in controls is notably skewed). The use of repeated-measures ANOVA for postoperative NRS scores is appropriate and addresses the prior reviewer concern about multiple comparisons.

The sample size calculation deserves more scrutiny than it has received across revision cycles. A minimum of 7 subjects per group to detect the primary effect is correct arithmetically, but the variance estimates were drawn directly from the very study (Pawan et al.) whose results the authors then nearly exactly replicate—this circularity inflates the apparent adequacy of 30 per group and should be discussed more explicitly as a limitation. Relatedly, the authors justify enrolling 30 per group on grounds of "precision, external validity, and attrition," but these rationale lack quantitative grounding. A brief sensitivity analysis or commentary on the trial's post-hoc power for secondary outcomes would strengthen this section considerably.

The regression analyses performed to adjust for baseline imbalances in BMI, religion, and ethnicity are a welcome addition. However, the manuscript does not report the actual regression coefficients, adjusted effect estimates, or confidence intervals in any table—it only references that regression was performed. These results should be presented explicitly, even in a supplementary table, to allow the reader to assess whether the observed imbalances materially affect the primary conclusions.

Limitations and Weaknesses

Several limitations are acknowledged by the authors but deserve amplification. First, the retrospective trial registration (PACTR202509579633417) remains a substantive concern that cannot be fully mitigated by explanation alone. Retrospective registration introduces the possibility of outcome reporting bias that prospective registration is designed to prevent. The explanation offered—administrative delays and internet access difficulties—is contextually plausible given the study setting, but the reader should weigh this caveat accordingly.

Second, partial blinding is a structural limitation with real implications for this trial. The anesthetists administering the block were aware of group allocation, and though pain scores were recorded by ward nurses using a standardized scale, the manuscript does not clearly describe whether those ward nurses were blinded to treatment assignment. If they were not, NRS scoring could be susceptible to expectation bias, particularly given the subjective nature of pain reporting. This should be stated more explicitly.

Third, the 24-hour NRS data are reported descriptively but not analyzed due to widespread rescue analgesia use in the control group—an entirely defensible decision. However, the resulting analytic gap leaves the manuscript unable to characterize the duration of the dexamethasone effect's decay and its convergence with the control group. A time-to-event analysis (e.g., survival analysis for analgesia duration) would have been more informative than a simple t-test of means and would have better handled the censoring inherent in rescue analgesia.

Fourth, the reference list contains several entries with incomplete bibliographic data. References 4, 7, 9, 10, 13, 14, 17, 20, 21, 22, 23, and 24 are missing journal names, volume numbers, and page ranges. Reference 12 (Suzuki et al., Local Anesthetics, Pharmacology and Physiology for Anesthesia, Elsevier, 2019) is cited in support of the claim that dexamethasone induces vasoconstriction—a mechanism more appropriately attributed to a primary dexamethasone pharmacology source. These should be corrected and verified for accuracy before publication.

Minor Points

The trial registration section on the title page contains an apparent typographic error: recruitment is listed as occurring between "September 23, 2025 and January 17, 2025"—a logically impossible date range. This presumably reflects a typo (2024, not 2025) but must be corrected, as it undermines confidence in manuscript proofreading.

The contribution statement contains a typo ("ollection" rather than "collection") that was present in the prior submission and has not been corrected in the revised version.

The Discussion section would benefit from a more explicit acknowledgment that all enrolled patients were ASA I—a notably low-risk, healthy cohort that may limit generalizability to the broader perioperative population, including diabetic or immunocompromised patients in whom dexamethasone's risk profile is more nuanced.

Summary

Overall, this manuscript addresses a meaningful clinical question in an underrepresented setting and reports large, internally consistent findings. The revisions undertaken across multiple review cycles have improved the manuscript's transparency and methodological reporting. The remaining concerns—particularly around regression result reporting, blinding of ward nurses, the registration timeline error, and incomplete references—are addressable and should be resolved before acceptance. I would recommend Major Revision.

**********

what does this mean?). If published, this will include your full peer review and any attached files.

If you choose “no”, your identity will remain anonymous but your review may still be made public.

**Do you want your identity to be public for this peer review?**  For information about this choice, including consent withdrawal, please see our Privacy Policy

Reviewer #1: No

Reviewer #5: No

Reviewer #6: No

**********

[NOTE: If reviewer comments were submitted as an attachment file, they will be attached to this email and accessible via the submission site. Please log into your account, locate the manuscript record, and check for the action link "View Attachments". If this link does not appear, there are no attachment files.]

To ensure your figures meet our technical requirements, please review our figure guidelines: https://journals.plos.org/plosone/s/figures

You may also use PLOS’s free figure tool, NAAS, to help you prepare publication quality figures: https://journals.plos.org/plosone/s/figures#loc-tools-for-figure-preparation.

NAAS will assess whether your figures meet our technical requirements by comparing each figure against our figure specifications.

# Author comment: Author response to Decision Letter 3

**DOI:** 10.1371/journal.pone.0338067.r006

17 Jun 2026

Reviewer #1:

We have added explicit reporting of Shapiro–Wilk and Levene’s tests to confirm statistical assumptions, and expanded regression reporting with adjusted effect estimates, confidence intervals, and a supplementary table.

Reviewer #5:

We appreciate the recognition of the clinical relevance of dexamethasone. The analgesics table has been proofread and clarified for consistency.

Reviewer #6:

We have incorporated a sensitivity analysis for sample size, added post‑hoc power commentary, clarified retrospective trial registration, and acknowledged partial blinding with potential expectation bias. We also explicitly note that all patients were ASA I, limiting generalizability, and explain why time‑to‑event analysis was not feasible, while recommending survival analysis in future larger studies. References and typographic errors have been corrected.

Attachment Submitted filename: Response_to_Reviewers_June2026.docx.docx

# Peer review: Decision Letter 3

**DOI:** 10.1371/journal.pone.0338067.r007

**Authors:** Robert Jeenchen Chen

8 Jul 2026

Dear Dr. Mengistu,

Please submit your revised manuscript by Aug 22 2026 11:59PM. If you will need more time than this to complete your revisions, please reply to this message or contact the journal office at plosone@plos.org. When you're ready to submit your revision, log on to https://www.editorialmanager.com/pone/ and select the 'Submissions Needing Revision' folder to locate your manuscript file.

- A letter that responds to each point raised by the academic editor and reviewer(s). You should upload this letter as a separate file labeled 'Response to Reviewers'.
- A marked-up copy of your manuscript that highlights changes made to the original version. You should upload this as a separate file labeled 'Revised Manuscript with Track Changes'.
- An unmarked version of your revised paper without tracked changes. You should upload this as a separate file labeled 'Manuscript'.

If you would like to make changes to your financial disclosure, please include your updated statement in your cover letter. Guidelines for resubmitting your figure files are available below the reviewer comments at the end of this letter.

If applicable, we recommend that you deposit your laboratory protocols in protocols.io to enhance the reproducibility of your results. Protocols.io assigns your protocol its own identifier (DOI) so that it can be cited independently in the future. For instructions see: https://journals.plos.org/plosone/s/submission-guidelines#loc-laboratory-protocols. Additionally, PLOS ONE offers an option for publishing peer-reviewed Lab Protocol articles, which describe protocols hosted on protocols.io. Read more information on sharing protocols at https://plos.org/protocols?utm_medium=editorial-email&utm_source=authorletters&utm_campaign=protocols.

As the corresponding author, your ORCID iD is verified in the submission system and will appear in the published article. PLOS supports the use of ORCID, and we encourage all coauthors to register for an ORCID iD and use it as well. Please encourage your coauthors to verify their ORCID iD within the submission system before final acceptance, as unverified ORCID iDs will not appear in the published article. *Only*  the individual author can complete the verification step; PLOS staff *cannot*  verify ORCID iDs on behalf of authors.

We look forward to receiving your revised manuscript.

Kind regards,

Robert Jeenchen Chen, MD, MPH, ChFC®, EA

Academic Editor

PLOS One

Journal Requirements:

1. If the reviewer comments include a recommendation to cite specific previously published works, please review and evaluate these publications to determine whether they are relevant and should be cited. There is no requirement to cite these works unless the editor has indicated otherwise.

Additional Editor Comments:

Revise

[Note: HTML markup is below. Please do not edit.]

Reviewer's Responses to Questions

**Comments to the Author**

Reviewer #1: (No Response)

Reviewer #7: (No Response)

Reviewer #8: (No Response)

**********

2. Is the manuscript technically sound, and do the data support the conclusions?

Reviewer #1: (No Response)

Reviewer #7: No

Reviewer #8: Yes

**********

3. Has the statistical analysis been performed appropriately and rigorously?

Reviewer #1: (No Response)

Reviewer #7: Yes

Reviewer #8: No

**********

4. Have the authors made all data underlying the findings in their manuscript fully available?

The PLOS Data policy

Reviewer #1: (No Response)

Reviewer #7: No

Reviewer #8: Yes

**********

5. Is the manuscript presented in an intelligible fashion and written in standard English?

Reviewer #1: (No Response)

Reviewer #7: Yes

Reviewer #8: No

**********

Reviewer #1: .........................................................................................................................................................

Reviewer #7: Abstract:

- method is incomplete and the inclusion criteria are not exactly clear especially age, ASA class and elective or emergency and also type of operation.

-primary and secondary outcomes are not mentioned.

- It is better that results write comparatively and the numerical values of intensity and time between the 2 groups should be mentioned.

introduction:

- It would better to use newer references and the contents should be cited from recent references.

Material and Methods:

- This item is not complete.

- The specification and manufacture company of usage drugs should be mentioned.

- The side effects including hypotension, bradycardia and respiratory depression should be defined and the method of managing them should be explained.

- The groups maybe not matched according to type of surgery(skeletal or soft tissue) and emergency or elective operation. These items should be mentioned.

- The technique of performing procedures must be fully described such as: patient positioning, procedure landmark and type of ultrasound-guided usage.

- How has the duration of operation been calculated. At what time and by whom was the onset of speed of the sensory and motor block assessed.

- Randomization and blindness should be completely explained.

- How was the postoperative pain management? such as: depend on NRS score, type of analgesic drugs and etc

- Did the patients receive any sedation during the operation? Have they had any failed block? Please fully explained and the Diagram consort should also be included.

Results:

- All results are not presented such as : side effects including hypotension. bradycardia, respiratory depression , nausea and vomiting etc

- Is there date available regarding analgesic usage by time interval of assessing? If there is, please attach it and provide explanation in the discussion.

- The way that results are expressed is not interesting, for example some results can be expressed as figure such as : the hemodynamic parameters

Discussion:

- The discussion is incomplete and scattered. The findings are not correctly described.

- It is advisable to explain and further discuss on the results regarding the pharmacokinetic and pharmacodynamic of dexamethasone such as : the onset and the mechanism of action, duration of action and its therapeutic effect.

References:

- Most of the references are quiet old and it would better to replace them with more recent ones.

Reviewer #8: “The Effect of Intravenous Dexamethasone in Supraclavicular Brachial Plexus Block at Halibet National Referral Hospital, Eritrea 2024 - 2025: A Randomized Clinical Trial”

The full title brings confusion in some way. Intravenous dexamethasone with SCBPB may be better.

Intravenous dexamethasone has been demonstrated to prolong analgesia similarly to perineural administration, which limits the novelty of this finding. However, this report does provide a comparison of the onset of supraclavicular brachial plexus block (SCBPB) and the sequential analgesic effects following upper limb surgery.

Abstract

The abstract needs an extensive revision to make it clear. Such as

Background:

4th line, This study aimed to evaluate the impact of adding intravenous dexamethasone on postoperative analgesia in conjunction with SCBPB for upper limb surgeries.

Methods

The 2nd line, Regarding the timing of performing SCBPB with 20 mL of 0.5% bupivacaine, the control group received 2 mL of normal saline intravenously, while the experimental group received the same intravenous volume containing 8 mg of dexamethasone.

**********

what does this mean?). If published, this will include your full peer review and any attached files.

If you choose “no”, your identity will remain anonymous but your review may still be made public.

**Do you want your identity to be public for this peer review?**  For information about this choice, including consent withdrawal, please see our Privacy Policy

Reviewer #1: No

Reviewer #7: No

Reviewer #8: No

**********

[NOTE: If reviewer comments were submitted as an attachment file, they will be attached to this email and accessible via the submission site. Please log into your account, locate the manuscript record, and check for the action link "View Attachments". If this link does not appear, there are no attachment files.]

To ensure your figures meet our technical requirements, please review our figure guidelines: https://journals.plos.org/plosone/s/figures

You may also use PLOS’s free figure tool, NAAS, to help you prepare publication quality figures: https://journals.plos.org/plosone/s/figures#loc-tools-for-figure-preparation.

NAAS will assess whether your figures meet our technical requirements by comparing each figure against our figure specifications.

# Author comment: Author response to Decision Letter 4

**DOI:** 10.1371/journal.pone.0338067.r008

22 Jul 2026

We believe these revisions address all outstanding concerns from Reviewer #7 and Reviewer #8. The manuscript has been revised and polished to meet the criteria of PLOS ONE. Most of the comments provided by the recent reviewers were already addressed in the previous cycle of revision, and we have highlighted where these clarifications are present. Given the extensive revisions and multiple independent reviews already completed, we respectfully request that the editor consider this version suitable for publication in PLOS ONE.

Attachment Submitted filename: Response_to_Reviewers_July2026.docx.docx

# Peer review: Decision Letter 4

**DOI:** 10.1371/journal.pone.0338067.r009

**Authors:** Robert Chen

28 Jul 2026

Dear Dr. Mengistu,

Thank you for submitting your manuscript to PLOS ONE. After careful consideration, we feel that it has merit but does not fully meet PLOS ONE’s publication criteria as it currently stands. Therefore, we invite you to submit a revised version of the manuscript that addresses the points raised during the review process.

Revise.

Please submit your revised manuscript by Sep 11 2026 11:59PM. If you will need more time than this to complete your revisions, please reply to this message or contact the journal office at plosone@plos.org. When you're ready to submit your revision, log on to https://www.editorialmanager.com/pone/ and select the 'Submissions Needing Revision' folder to locate your manuscript file.

Please include the following items when submitting your revised manuscript:

- A letter that responds to each point raised by the academic editor and reviewer(s). You should upload this letter as a separate file labeled 'Response to Reviewers'.
- A marked-up copy of your manuscript that highlights changes made to the original version. You should upload this as a separate file labeled 'Revised Manuscript with Track Changes'.
- An unmarked version of your revised paper without tracked changes. You should upload this as a separate file labeled 'Manuscript'.

If you would like to make changes to your financial disclosure, please include your updated statement in your cover letter. Guidelines for resubmitting your figure files are available below the reviewer comments at the end of this letter.

If applicable, we recommend that you deposit your laboratory protocols in protocols.io to enhance the reproducibility of your results. Protocols.io assigns your protocol its own identifier (DOI) so that it can be cited independently in the future. For instructions see: https://journals.plos.org/plosone/s/submission-guidelines#loc-laboratory-protocols. Additionally, PLOS ONE offers an option for publishing peer-reviewed Lab Protocol articles, which describe protocols hosted on protocols.io. Read more information on sharing protocols at https://plos.org/protocols?utm_medium=editorial-email&utm_source=authorletters&utm_campaign=protocols.

As the corresponding author, your ORCID iD is verified in the submission system and will appear in the published article. PLOS supports the use of ORCID, and we encourage all coauthors to register for an ORCID iD and use it as well. Please encourage your coauthors to verify their ORCID iD within the submission system before final acceptance, as unverified ORCID iDs will not appear in the published article. *Only*  the individual author can complete the verification step; PLOS staff *cannot*  verify ORCID iDs on behalf of authors.

We look forward to receiving your revised manuscript.

Kind regards,

Robert Jeenchen Chen, MD, MPH, ChFC®, EA

Academic Editor

PLOS One

Journal Requirements:

If the reviewer comments include a recommendation to cite specific previously published works, please review and evaluate these publications to determine whether they are relevant and should be cited. There is no requirement to cite these works unless the editor has indicated otherwise.

Please review your reference list to ensure that it is complete and correct. If you have cited papers that have been retracted, please include the rationale for doing so in the manuscript text, or remove these references and replace them with relevant current references. Any changes to the reference list should be mentioned in the rebuttal letter that accompanies your revised manuscript. If you need to cite a retracted article, indicate the article’s retracted status in the References list and also include a citation and full reference for the retraction notice.

[Note: HTML markup is below. Please do not edit.]

Reviewers' comments:

Reviewer's Responses to Questions

**Comments to the Author**

Reviewer #1: All comments have been addressed

Reviewer #7: All comments have been addressed

Reviewer #8: (No Response)

**********

2. Is the manuscript technically sound, and do the data support the conclusions?

Reviewer #1: Yes

Reviewer #7: Yes

Reviewer #8: Partly

**********

3. Has the statistical analysis been performed appropriately and rigorously?

Reviewer #1: Yes

Reviewer #7: Yes

Reviewer #8: Yes

**********

4. Have the authors made all data underlying the findings in their manuscript fully available?

The PLOS Data policy

Reviewer #1: Yes

Reviewer #7: Yes

Reviewer #8: Yes

**********

5. Is the manuscript presented in an intelligible fashion and written in standard English?

Reviewer #1: Yes

Reviewer #7: Yes

Reviewer #8: No

**********

Reviewer #1: All comments have been addressed. I have nothing else to raise.,..........,,... ..........

Reviewer #7: please revised and responded to all recommendation and highlight them in the text. I can not see all revision in the text.

Reviewer #8: As a reviewer, my role is to assist authors in clearly presenting their research. Unfortunately, the revised version does not meet this objective.

Below are my suggestions:

Background: Postoperative pain has a significant impact on recovery and healthcare costs. While effective regional anesthesia provides both anesthesia and analgesia, its effects are often of limited duration. Previous studies indicate that intravenous dexamethasone may enhance and prolong analgesia when used with regional blocks, yet reports on its addition to supraclavicular brachial plexus block (SCBPB) for upper limb surgeries are limited. We hypothesized that intravenous dexamethasone would extend postoperative analgesia following SCBPB.

Method: A randomized clinical trial was conducted at Halibet National Referral Hospital in Eritrea, involving 60 patients undergoing upper limb surgery who received SCBPB with 20 ml of 0.5% bupivacaine. Concurrently, the control group received 2 ml of normal saline, while the experimental group received 2 ml (8 mg) of intravenous dexamethasone. Pain scores, time to first rescue analgesia, and analgesic consumption were recorded. Data were analyzed using SPSS v26.

**********

what does this mean?). If published, this will include your full peer review and any attached files.

If you choose “no”, your identity will remain anonymous but your review may still be made public.

**Do you want your identity to be public for this peer review?**  For information about this choice, including consent withdrawal, please see our Privacy Policy

Reviewer #1: No

Reviewer #7: No

Reviewer #8: No

**********

[NOTE: If reviewer comments were submitted as an attachment file, they will be attached to this email and accessible via the submission site. Please log into your account, locate the manuscript record, and check for the action link "View Attachments". If this link does not appear, there are no attachment files.]

To ensure your figures meet our technical requirements, please review our figure guidelines: https://journals.plos.org/plosone/s/figures

You may also use PLOS’s free figure tool, NAAS, to help you prepare publication quality figures: https://journals.plos.org/plosone/s/figures#loc-tools-for-figure-preparation.

NAAS will assess whether your figures meet our technical requirements by comparing each figure against our figure specifications.

# Author comment: Author response to Decision Letter 5

**DOI:** 10.1371/journal.pone.0338067.r010

3 Aug 2026

We thank all the reviewers for their constructive comments and suggestions.

Attachment Submitted filename: Response_to_Reviewers_August2026.docx.docx

# Peer review: Decision Letter 5

**DOI:** 10.1371/journal.pone.0338067.r011

**Authors:** Robert Jeenchen Chen

11 Aug 2026

The Effect of Intravenous Dexamethasone in Supraclavicular Brachial Plexus Block at Halibet National Referral Hospital, Eritrea 2024 - 2025: A Randomized Clinical Trial

PONE-D-25-60591R5

Dear Dr. Mengistu,

We’re pleased to inform you that your manuscript has been judged scientifically suitable for publication and will be formally accepted for publication once it meets all outstanding technical requirements.

Within one week, you’ll receive an e-mail detailing the required amendments. When these have been addressed, you’ll receive a formal acceptance letter and your manuscript will be scheduled for publication.

An invoice will be generated when your article is formally accepted. Please note, if your institution has a publishing partnership with PLOS and your article meets the relevant criteria, all or part of your publication costs will be covered. Please make sure your user information is up-to-date by logging into Editorial Manager at Editorial Manager® and clicking the ‘Update My Information' link at the top of the page. For questions related to billing, please contact billing support.

If your institution or institutions have a press office, please notify them about your upcoming paper to help maximize its impact. If they’ll be preparing press materials, please inform our press team as soon as possible -- no later than 48 hours after receiving the formal acceptance. Your manuscript will remain under strict press embargo until 2 pm Eastern Time on the date of publication. For more information, please contact onepress@plos.org.

Kind regards,

Robert Jeenchen Chen, MD, MPH, ChFC®, EA

Academic Editor

PLOS One

Additional Editor Comments (optional):

Reviewers' comments:

Reviewer's Responses to Questions

**Comments to the Author**

Reviewer #1: (No Response)

Reviewer #7: All comments have been addressed

Reviewer #8: (No Response)

**********

2. Is the manuscript technically sound, and do the data support the conclusions?

Reviewer #1: (No Response)

Reviewer #7: Yes

Reviewer #8: (No Response)

**********

3. Has the statistical analysis been performed appropriately and rigorously?

Reviewer #1: (No Response)

Reviewer #7: Yes

Reviewer #8: (No Response)

**********

4. Have the authors made all data underlying the findings in their manuscript fully available?

The PLOS Data policy

Reviewer #1: (No Response)

Reviewer #7: Yes

Reviewer #8: (No Response)

**********

5. Is the manuscript presented in an intelligible fashion and written in standard English?

Reviewer #1: (No Response)

Reviewer #7: Yes

Reviewer #8: (No Response)

**********

Reviewer #1: ..............................................................................................................................

Reviewer #7: Please mention the specification of drugs usage(Bupivacaine and Dexamethasone) including volume of vial, mg per ml, and the company and country of manufacture.

Reviewer #8: (No Response)

**********

what does this mean?). If published, this will include your full peer review and any attached files.

If you choose “no”, your identity will remain anonymous but your review may still be made public.

**Do you want your identity to be public for this peer review?**  For information about this choice, including consent withdrawal, please see our Privacy Policy

Reviewer #1: No

Reviewer #7: No

Reviewer #8: No

**********

# Editor report: Acceptance letter

**DOI:** 10.1371/journal.pone.0338067.r012

**Authors:** Robert Jeenchen Chen

PONE-D-25-60591R5

PLOS One

Dear Dr. Mengistu,

I'm pleased to inform you that your manuscript has been deemed suitable for publication in PLOS One. Congratulations! Your manuscript is now being handed over to our production team.

At this stage, our production department will prepare your paper for publication. This includes ensuring the following:

* All references, tables, and figures are properly cited

* All relevant supporting information is included in the manuscript submission,

* There are no issues that prevent the paper from being properly typeset

You will receive further instructions from the production team, including instructions on how to review your proof when it is ready. Please keep in mind that we are working through a large volume of accepted articles, so please give us a few days to review your paper and let you know the next and final steps.

Lastly, if your institution or institutions have a press office, please let them know about your upcoming paper now to help maximize its impact. If they'll be preparing press materials, please inform our press team within the next 48 hours. Your manuscript will remain under strict press embargo until 2 pm Eastern Time on the date of publication. For more information, please contact onepress@plos.org.

You will receive an invoice from PLOS for your publication fee after your manuscript has reached the completed accept phase. If you receive an email requesting payment before acceptance or for any other service, this may be a phishing scheme. Learn how to identify phishing emails and protect your accounts at https://explore.plos.org/phishing.

If we can help with anything else, please email us at customercare@plos.org.

Thank you for submitting your work to PLOS One and supporting open access.

Kind regards,

PLOS One Editorial Office Staff

on behalf of

Dr. Robert Jeenchen Chen

Academic Editor

PLOS One
